# ﻿Chemical clues to infection: A pilot study on the differential secondary metabolite production during the life cycle of selected *Cordyceps* species

**DOI:** 10.3897/imafungus.16.172651

**Published:** 2025-12-12

**Authors:** Esteban Charria Girón, Rita Toshe, Artit Khonsanit, Noppol Kobmoo, Papichaya Kwanthong, Tatiana E. Gorelik, Janet Jennifer Luangsa-ard, Sherif S. Ebada, Marc Stadler

**Affiliations:** 1 Department Microbial Drugs, Helmholtz Centre for Infection Research, Inhoffenstraße 7, 38124 Braunschweig, Germany; 2 Bioinformatics Group, Wageningen University & Research, Droevendaalsesteeg 1, 6708 PB Wageningen, Netherlands; 3 67 Moo 9 Ban Huai, Muang Nong Hang, Kuchinarai, Kalasin, Thailand; 4 Plant Microbe Interaction Research Team, BIOTEC, National Science and Technology Development Agency (NSTDA) 111 Thailand Science Park, Phahonyothin Road, Khlong Nueng, Khlong Luang, Pathum Thani, 12120, Thailand; 5 Department Structure and Function of Proteins, Helmholtz Centre for Infection Research (HZI), 38124 Braunschweig, Germany; 6 Department Microbial Natural Products, Helmholtz-Institute for Pharmaceutical Research Saarland (HIPS), 66123 Saarbrücken, Germany; 7 Ernst Ruska-Centre for Microscopy and Spectroscopy with Electrons (ER-C), Forschungszentrum Jülich, 52425 Jülich, Germany; 8 Department of Pharmacognosy, Faculty of Pharmacy, Ain Shams University, 11566 Cairo, Egypt; 9 Institute of Microbiology, Technische Universität Braunschweig, Spielmannstraße 7, 38106 Braunschweig, Germany

**Keywords:** *
Cordycipitaceae
*, Entomopathogenic fungi, host-colonization, metabolomics, virulence

## Abstract

*Cordyceps* species are widespread entomopathogens and promising biocontrol agents that produce diverse secondary metabolites, yet the roles of these molecules during the infection process remain unclear. To interpret how fungal chemistry contributes to host colonization, we compared the metabolomes and virulence traits of two strains of phylogenetically distinct *Cordyceps* species (*C. javanica* and *C. blackwelliae*) and assessed their effects on beet armyworms (*fungiSpodoptera exigua*). Virulence assays revealed species-dependent pathogenicity, with *C. javanica* showing the highest virulence. Combining untargeted metabolomics, feature-based molecular networking (FBMN), 3D electron-diffraction crystallography and comprehensive 1D/2D NMR, we gained insights into their metabolomic traits. For instance, *C. javanica* displayed notable beauveriolide diversity, including three previously undescribed derivatives (**1**–**3**), while *C. blackwelliae* produced mainly diketopiperazines *in vitro.* The FBMN results revealed putative beauveriolide analogs in the *C. blackwelliae* extracts, unlike the cadaver analysis, revealing beauvericins in infected corpses. Remarkably, the crude extracts obtained from authentic insect cadavers contained beauveriolides and beauvericins, providing *in vivo* chemical evidences of their production during infection for the first time. Moreover, bioassays with purified compounds showed that insecticidal activity cannot be attributed across all beauveriolides but depends on amino-acid composition, implying multifunctional roles beyond direct toxicity. Altogether, these results reveal context-dependent metabolic reprogramming and species-specific chemical strategies in entomopathogenic fungi, with implications for microbial ecology, host specificity, and the rational development of fungal biocontrol agents. The results of this study also give rise to the need for more intensified study on the chemical composition of the insect cadavers that are colonized by other entomopathogens.

## ﻿Introduction

The genus *Cordyceps (Hypocreales)* includes fungi renowned for their ability to parasitize insects, a unique trait defining their role as entomopathogens. Historically, fungi associated with insects and arachnids featuring conspicuous stromata were classified in this genus (Shrestha et al. 2014). However, successive taxonomic revisions based on molecular data have led to their reclassification into various families within the *Hypocreales* order with up to 23 different genera currently accepted within the *Cordycipitaceae* (Sung et al. 2007; Kepler et al. 2017; Shrestha et al. 2017; Wang et al. 2020). These revisions have significantly broadened our understanding of the diversity within these families, providing a robust foundation for the study of their ecology and derived applications. Preliminary correlations based on phylogenetic information could be found between certain genera and host/substrate specificity, suggesting their ability to colonize and effectively target specific insect hosts (Kobmoo et al. 2023; Khonsanit et al. 2024). This unique parasitic behavior has been exploited to develop specific biocontrol agents in agriculture.

Moreover, species of *Cordyceps* and *Ophiocordyceps* are of significant value in traditional Chinese medicine, where both the fungus and the dead insect host are consumed together. This duo mirrors the philosophical yin and yang concept, representing the balance between life and death and natural dualities that together restore harmony (Paterson 2008). These combinations have long been employed to treat illnesses, including respiratory and liver disorders, cardiovascular diseases, low libido, hyperlipidemia, and chronic kidney disease (Paterson 2008). In fact, currently the global market for these applications within traditional Chinese medicine is estimated to be worth up to USD 1.5 billion (Niego et al. 2023).

*Cordyceps* species are known as prolific producers of bioactive secondary metabolites. These compounds might be involved in suppressing host defenses or manipulating host physiology, yet their exact role in the infection process remains poorly understood (Yin et al. 2020; Sun et al. 2022; Kobmoo et al. 2024). Despite the potential involvement of these metabolites in virulence, this aspect of *Cordyceps* chemical biology is still largely understudied. In contrast, these compounds might prove useful in the development of therapeutic treatments due to their multifaceted roles and diverse biological properties (Helaly et al. 2019; Toshe et al. 2024). The current pilot study describes the metabolome and virulence traits of *C. javanica* and *C. blackwelliae*, two strains of insect-associated fungi that were selected for their distinct ecological roles and potential as biocontrol agents. By combining state-of-the-art metabolomics, classical chemical screening, and virulence assays, we aimed to identify key metabolites involved in the infection process and evaluate their suitability for pest control applications.

## ﻿Materials and methods

### ﻿General experimental procedures

Optical rotation was measured at 20 °C on an MCP-150 polarimeter (Anton-Paar Opto Tec GmbH, Seelze, Germany). UV/Vis spectra were recorded on a UV-2450 spectrophotometer (Shimadzu, Kyoto, Japan). NMR spectra were acquired using an Avance III 500 spectrometer (Bruker, Billerica, MA, USA; ^1^H NMR at 500 MHz, ^13^C NMR at 125 MHz), with samples in DMSO-*d*_6_. High-resolution electrospray ionization mass spectra (HR-ESI-MS) were acquired using an Agilent 1200 Infinity Series HPLC-UV system (Agilent Technologies, Santa Clara, CA, USA) with a C_18_ Acquity UPLC BEH column (50 × 2.1 mm, 1.7 μm; Waters, Milford, MA, USA). The mobile phases were: solvent A (H_2_O + 0.1% formic acid) and solvent B (acetonitrile + 0.1% formic acid). The gradient started at 5% B for 0.5 min, increasing to 100% B over 19.5 min, holding at 100% B for 5 min, with flow rate of 0.6 mL/min, with UV/Vis detection at 190–600 nm. The system was connected to a time-of-flight mass spectrometer (ESI-TOF-MS, maXis, Bruker, Billerica, MA, USA; scan range 100–2500 *m*/*z*, rate 2 Hz, capillary voltage 4500 V, dry temperature 200 °C).

### ﻿Fungal material

*Cordyceps blackwelliae* (BCC37653) was isolated from a lepidopteran pupa found on the underside of a leaf in Khao Yai National Park, while *C. javanica*BCC 79245 (TBRC 7259) and BCC 82944 (TBRC 7262) were isolated from lepidopteran larvae in Nam Nao National Park and Wang Takrai Waterfalls, respectively. Pure cultures of *C. blackwelliae* and *C. javanica* have been previously deposited in the BIOTEC culture collection (BCC) (Mongkolsamrit et al. 2018; Helaly et al. 2019). Phylogenetic analyses based on ITS and *tef* sequences (GenBank accession numbers: *ITS* = PP709053, *tef* = PP735442) confirmed that BCC 37653 was nested with the type species of *C. blackwelliae* TBRC 7257 (Mongkolsamrit et al. 2018).

### ﻿Virulence assays

Fungal virulence was evaluated against beet armyworm (*fungiSpodoptera exigua*, Lepidoptera), using an established protocol at BIOTEC. The representative strains were grown on PDA at 25 °C until sporulation (ranging from one to four weeks). Spores were collected into 1 mL sterile water, counted, and then adjusted to a concentration of 10^8^ spores/mL. For each assay, beet armyworms were injected with 3 µL of spore suspension (n = 30 insects per strain, divided into three independent replicates of 10 individuals each). Mortality was monitored daily for 7 days and categorized as total mortality (TM), any dead insect, or mycelium-associated mortality (MM), dead insects showing visible external mycelial outgrowth. Mortality rate was calculated per replicate as the number of dead insects (TM or MM) divided by the total number of insects in each replicate.

To evaluate the virulence of purified compounds against beet armyworm larvae, beauveriolides I (**4**) and M (**5**) were selected based on availability, and cytochalasin D served as a positive control. Compounds were dissolved in DMSO and serially diluted to give concentrations ranging from 40 to 0.625 µg/mL. Larvae were injected with 0.5 µL of each dilution (n = 6 per concentration), maintained at 25 °C for one week, and monitored daily to assess mortality. Saline (8.5% NaCl) and DMSO served as negative controls.

### ﻿Metabolomics studies

To evaluate the production of secondary metabolites, *C. blackwelliae* and *C. javanica* were cultured in yeast malt liquid medium (YM 6.3; 10 g/L malt extract, 4 g/L D-glucose, 4 g/L yeast extract, pH 6.3 before autoclaving). Strains were first grown on YM agar and after sufficient growth, five plugs (7 mm diameter) were transferred into flasks containing 100 mL of SMYA medium and incubated at 25 °C with shaking at 220 rpm for 7 days. For screening, 3 mL aliquots of each seed culture were added to 500 mL flasks containing 200 mL of production medium and incubated at 23 °C and 140 rpm. Glucose levels were monitored daily, and cultures were harvested three days post-glucose depletion. Supernatant and mycelium were separated by vacuum filtration and processed separately. The supernatant was extracted twice with ethyl acetate, the combined organic phases were evaporated under reduced pressure. The mycelia were soaked in acetone, ultrasonicated, filtered, and the obtained acetone solution was evaporated, dispersed in water, and processed in a similar manner to the supernatant.

After the detection of diverse molecules, we focused on exploring beauveriolide diversity. *Cordyceps javanica* exhibited the highest yield and chemical diversity. Therefore, both *C. javanica* strains were cultured in five additional liquid media (ZM ½, Q6 ½, GG1, Supermalt, and MMK) and two solid media (BRFT and V+YES). Cultivation and extraction for the solid media followed the same procedures used for YM and liquid cultures, with solid cultures treated in the same manner as mycelia from liquid media as previously described (Helaly et al. 2019; Toshe et al. 2024).

To investigate the presence of secondary metabolites in insect host corpses, a tissue segment was excised from the interface between the insect body and the emerging fruiting body or visible mycelia. The tissue was extracted twice with 1 mL of acetone:methanol (1:1) by vigorous shaking followed by ultrasonication. Combined extracts were filtered, dried under reduced pressure, and reconstituted in 50 µL of DMSO for analysis. All crude extracts were analyzed using ultrahigh-performance liquid chromatography coupled with diode array detection and ion mobility tandem mass spectrometry (UHPLC-DAD-IM-MS/MS) under previously established instrumental setting conditions and processed following our established protocols (Pfütze et al. 2024; Harms et al. 2024).

### ﻿Scaled-up cultivation of *C. javanica* and purification of beauveriolides

Cultivation of *C. javanica*BCC 82944 was scaled up in GG1 (4 L). Mycelial and supernatant extracts showed similar LC-MS profiles and were combined (20 g). A liquid-liquid pre-separation was performed using *n*-heptane, methanol, and water. A 9:1 methanol-to-water solution was mixed with an equal volume of *n*-heptane in a separatory funnel, resulting in two immiscible phases and a precipitate in the methanol-water phase. The precipitate was collected by filtration (Fraction I, 6.0 g), then methanol-water phase corresponded to Fraction II (12.6 g), and the *n*-heptane phase to Fraction III (1.4 g).

Fraction I was purified using preparative HPLC on a Büchi Pure C-850 FlashPrep system (Büchi Labortechnik GmbH; Essen, Germany) using a Gemini C_18_ column (250 × 50 mm, 10 μm; Phenomenex^®^, Torrance, CA, USA). The mobile phase consisted of deionized water with 0.1% formic acid (solvent A) and acetonitrile (MeCN) with 0.1% formic acid (solvent B) at pH 2.5. The flow rate was set at 30 mL/min, and UV detection was recorded at 210, 254, 300, and 350 nm. For each run, 300 mg aliquot was injected (10 runs) and the separation was performed using a gradient from 30% to 55% B in 12 minutes, 55% to 85% B in 30 minutes, from 85% to 100% B in 10 minutes and ended with an isocratic elution at 100% B for 10 min. A total of six compounds were obtained: **3** (1.2 mg, *t*_R_ = 35 min), **5** (5.3 mg, *t*_R_ = 42 min), **1** (3.2 mg, *t*_R_ = 45 min), **4** (14.1 mg, *t*_R_ = 47 min), **6** (6.3 mg, *t*_R_ = 56 min) and **2** (1.7 mg, *t*_R_ = 54 min). Fractions II was purified using the same system and column but with the following gradient: 40% to 100% in 60 minutes and ended with isocratic elution at 100% B for 10 min. This procedure afforded **7** (6.9 mg, *t*_R_ = 62 min), **4** (712.7 mg, *t*_R_ = 42 min) and **6** (19.9 mg, *t*_R_ = 56 min).

### ﻿Scaled-up cultivation of *C. blackwelliae* and purification of diketopiperazines

To investigate the metabolites of *C. blackwelliae*BCC 37653, a scale-up cultivation was conducted on YM medium. Five mycelial plugs of fully-grown YMA plates were transferred to 500-mL flasks containing 200 mL of YM medium (10 L in total), which were incubated at 23 °C and 140 rpm. Glucose was monitored daily, and cultures were harvested three days after glucose depletion. Mycelia and supernatant were extracted following the same protocol used for the *C. javanica* scale-up, yielding 200 and 300 mg of crude extract for the mycelia and supernatant, respectively. Crude extracts were purified on a Gilson PLC 2250 preparative HPLC system (Gilson, Middleton, WI, USA) using a Gemini C_18_ column (250 × 50 mm, 10 μm; Phenomenex^®^, Torrance, CA, USA). Mobile phases and detection parameters were the same as above despite using the following gradient: from 35% B to 100% B in 75 minutes and ended with isocratic elution at 100% B for 10 minutes. This resulted in the isolation of compound **10** (3.2 mg, *t*_R_ = 26 min), **9** (2.8 mg, *t*_R_ = 27 min), **8** (5.9 mg, *t*_R_ = 28 min), **12** (0.48 mg, *t*_R_ = 47 min) and **11** (0.57 mg, *t*_R_ = 52 min).

*Beauveriolide Q* (**1**): White powder; UV-Vis (MeOH): λ_max_ 225; HR-ESI-MS: *m/z* 527.3226 [M+H]^+^ (calcd. 527.3228 for C_29_H_43_N_4_O_5_^+^), 549.3046 [M+Na]^+^ (calcd. 549.6574 for C_29_H_42_N_4_NaO_5_^+^); *t*_R_ = 11.64 min (LR-ESI-MS); C_29_H_42_N_4_O_5_ (526.32 g mol^−1^).

*Beauveriolide R* (**2**): White powder; UV-Vis (MeOH): λ_max_ 226; NMR data (^1^H: 500 MHz, ^13^C: 125 MHz, DMSO-*d*_6_) see Table 1; HR-ESI-MS: *m/z* 555.3540 [M+H]^+^ (calcd. 555.3541 for C_31_H_47_N_4_O_5_^+^), 577.3359 [M+Na]^+^ (calcd. 577.7105 for C_31_H_46_N_4_NaO_5_^+^); *t*_R_ = 13.19 min (LR-ESI-MS); C_31_H_46_N_4_O_5_ (554.35 g mol^−1^).

**Table 1.** 1D (^1^H and ^13^C) NMR data of **2**.

pos.	δ_C_^a^ type	δ_H_^b^ (multi, J[Hz])
**HDA**		
1	170.2, CO	
2	35.4, CH_2_	*α* 2.31 dd (14.0, 8.8)
		*β* 2.44 dd (14.0, 4.4)
3	75.8, CH	4.84 dt (10.2, 5.3)
4	34.8, CH	2.07 (m)
5	30.8, CH_2_	*α* 1.02 m; *β* 1.33 m
6	26.5, CH_2_	*α* 1.16 m; *β* 1.31 m
7	29.0, CH_2_	1.23 overlapped
8	31.2, CH_2_	1.23 overlapped
9	22.1, CH_2_	1.24 overlapped
10	14.0, CH_3_	0.84 t (6.9)
11	15.5, CH_3_	0.80 d (6.9)
**Trp**		
1	171.4, CO	
2	56.0, CH	4.20 q (7.7)
3	25.7, CH_2_	*α* 3.03 dd (14.5, 8.0)
		*β* 3.11 dd (14.5, 7.3)
4	109.8, C	
4a	127.1, C	
5	118.3, CH	7.50 d (7.5)
6	118.4, CH	6.97 t (7.5)
7	121.0, CH	7.06 t (7.5)
8	111.4, CH	7.32 d (7.5)
8a	136.1, C	
9-N*H*	–	10.86 d (2.5)
10	123.6, CH	7.11 d (2.5)
N*H*	–	8.42 d (7.6)
**Ala**		
1	170.8, CO	
2	48.4, CH	3.90 p (6.9)
3	15.6, CH_3_	1.12 d (6.9)
N*H*	–	8.33 d (7.4)
**Leu**		
1	169.6, CO	
2	52.0, CH	4.39 q (8.0)
3	40.8, CH_2_	1.43 t (7.4)
4	24.4, CH	1.49 m
5	22.0, CH_3_	0.85 d (6.5)
6	22.2, CH_3_	0.87 d (6.5)
N*H*	–	7.19 d (9.2)

Measured in DMSO-*d*_6_ at *^a^* 125 MHz for ^13^C and *^b^* 500 MHz for ^1^H.

*Beauveriolide S* (**3**): Colorless oil; UV-Vis (MeOH): λ_max_ 225; HR-ESI-MS: *m/z* 412.2809 [M+H]^+^ (calcd. 412.2806 for C_21_H_38_N_3_O_5_^+^), 434.2625 [M+Na]^+^ (calcd. 434.5254 for C_21_H_37_N_3_NaO_5_^+^); *t*_R_ = 9.78 min (LR-ESI-MS); C_21_H_37_N_3_O_5_ (411.27 g mol^−1^).

### ﻿Single crystal structure via 3D electron diffraction of beauveriolide Q

Dry material was suspended in *n*-hexane and a drop of the suspension was placed onto a holey-carbon-coated copper TEM grid. Grids were air-dried and excess solvent soaked up using filter paper. Grids were transferred into a TEM at room temperature and cooled to liquid nitrogen temperature directly in the TEM vacuum. Electron diffraction data were collected using a Thermo Fisher GLACIOS TEM operating at 200 kV employing the EPU-D (Thermo Fisher) module. Diffraction patterns were recorded in nanodiffraction mode with an effective probe beam size on the sample of 1 micron.

Data were recorded in MRC format and converted to 16-bit TIFFs using the MRC2TIFF (10.5281/zenodo.7936068). TIFF sequences were then processed in PETS2 (Palatinus et al. 2019). The best-performing dataset was selected for structure analysis. Unit cell parameters were: a = 16.5864 Å, b = 5.1703 Å, c = 17.3397 Å, α = 90.000°, β = 94.011°, γ = 90.000°, with a volume of 1483 Å, corresponding to a monoclinic unit cell. Extinctions corresponding to a *2_1_* screw axis along the b-axis were detected, with no further extinctions observed, indicating the *P2_1_* space group (No. 4). The expected molecular volume was 719.74 Å³ (Hofmann 2002), corresponding to two molecules in the unit cell. The structure was solved using SHELXD and kinematically refined with SHELXL (Sheldrick 2015). The absolute configuration was determined during the dynamical refinement in JANA (Palatinus et al. 2015; Petrǐcěk et al. 2023). Further crystallographic details are provided in the Suppl. material 1.

### ﻿Antimicrobial and cytotoxic assays

Serial dilution assays were performed in 96-well microtiter plates to determine the minimum inhibitory concentrations (MICs) against a panel of yeasts, filamentous fungi, and bacteria, as well as the half-maximal inhibitory concentrations (IC_50_) against human cell lines, following our previously described protocol (Charria-Girón et al. 2023).

## ﻿Results and discussion

### ﻿Virulence and chemical traits from studied *Cordyceps* strains

**Figure 1.**
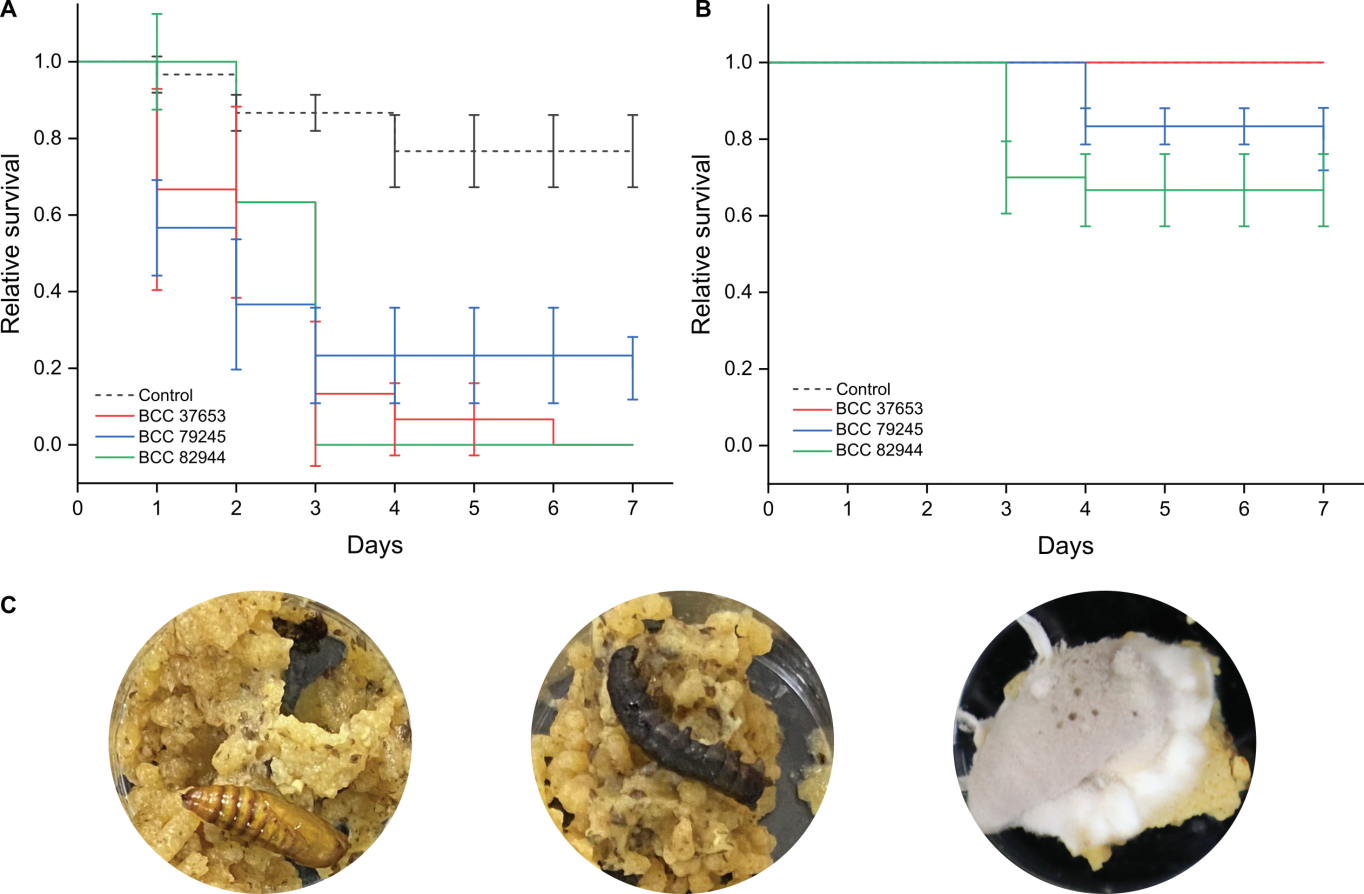
Virulence of *Cordyceps* spp., *C. blackwelliae*BCC 37653, *C. javanica*BCC 79245, and BCC 82944 against the beet armyworm (*fungiSpodoptera exigua*, *Lepidoptera*) observed over a 7-day period. **A** Relative survival considering total mortality (TM). **B** Relative survival considering mycelium-associated mortality (MM). Error bars denote the standard deviation across three independent biological replicates. **C** From left to right: larva turned into pupa, deceased insect without visible mycelia, and deceased insect with visible mycelia.

The virulence of three *Cordyceps* strains was evaluated against the beet armyworm (*fungiSpodoptera exigua*, *Lepidoptera*). *Cordyceps blackwelliae* and *C. javanica* strains were originally derived from a *Lepidoptera* pupae and larvae, respectively. Two types of insect mortality were considered: (TM) total mortality encompassing all deceased individuals, and (MM) mycelium-associated mortality representing dead insects exhibiting visible mycelia. This distinction was made to identify distinct virulence strategies. Entomopathogens are known to produce secondary metabolites, particularly mycotoxins (Schrank and Vainstein 2010; Cito et al. 2016), inside their host to cause death. Fungal growth and toxin production must be carefully balanced, as premature host death can prevent accumulation of nutrients required for development and sporulation (Boomsma et al. 2014). After insects are colonized and dead, fungi sporulate and disperse, processes that require favorable conditions and lytic activity to breach the cuticle from within (Charnley 2003). Therefore, visible mycelial outgrowth might indicate moderate toxin production combined with tissue-degrading enzymatic activity. Conversely, the absence of external mycelia suggests rapid, systemic killing driven by metabolites acting at the cellular level. From the two *C. javanica* strains, BCC 82944 was the most virulent, achieving 100% TM by day 3, while BCC 79245 caused an average TM of 80%, with both causing MM only around 20 to 40% (Fig. 1A). *Cordyceps blackwelliae* displayed an intermediate virulence with TM up to around 90% by day 3, but failed to produce visible mycelial outgrowth even after seven days (Fig. 1B). The evaluated strains caused high TM but differed in external mycelial development, with *C. javanica* seemingly producing more visible mycelia than *C. blackwelliae*. Observations of early host death suggest the production of active and host-targeted molecules, while differences in mycelial development likely reflect species-specific differences in metabolite repertoires and infection strategies.

Building on the hypothesis that secondary metabolites contribute to host infection, we decided to investigate their metabolome. However, it is noteworthy that metabolites produced during infection may differ from those observed under axenic cultivation. We cultured each fungus in yeast malt (YM) liquid medium until glucose was depleted. The obtained crude extracts were analyzed using ultrahigh-performance liquid chromatography coupled with diode array detection and ion mobility tandem mass spectrometry (UHPLC-DAD-IM-MS/MS), and raw data were pre-processed with MetaboScape (Charria-Girón et al. 2023). Features detected in blanks were excluded, and the resulting features were dereplicated against our in-house spectral library. While *C. javanica* was expected to produce beauveriolide-type cyclodepsipeptides and asperfurans (Helaly et al. 2019), *C. blackwelliae* was to the best of our knowledge never studied for secondary metabolite production.

**Figure 2.**
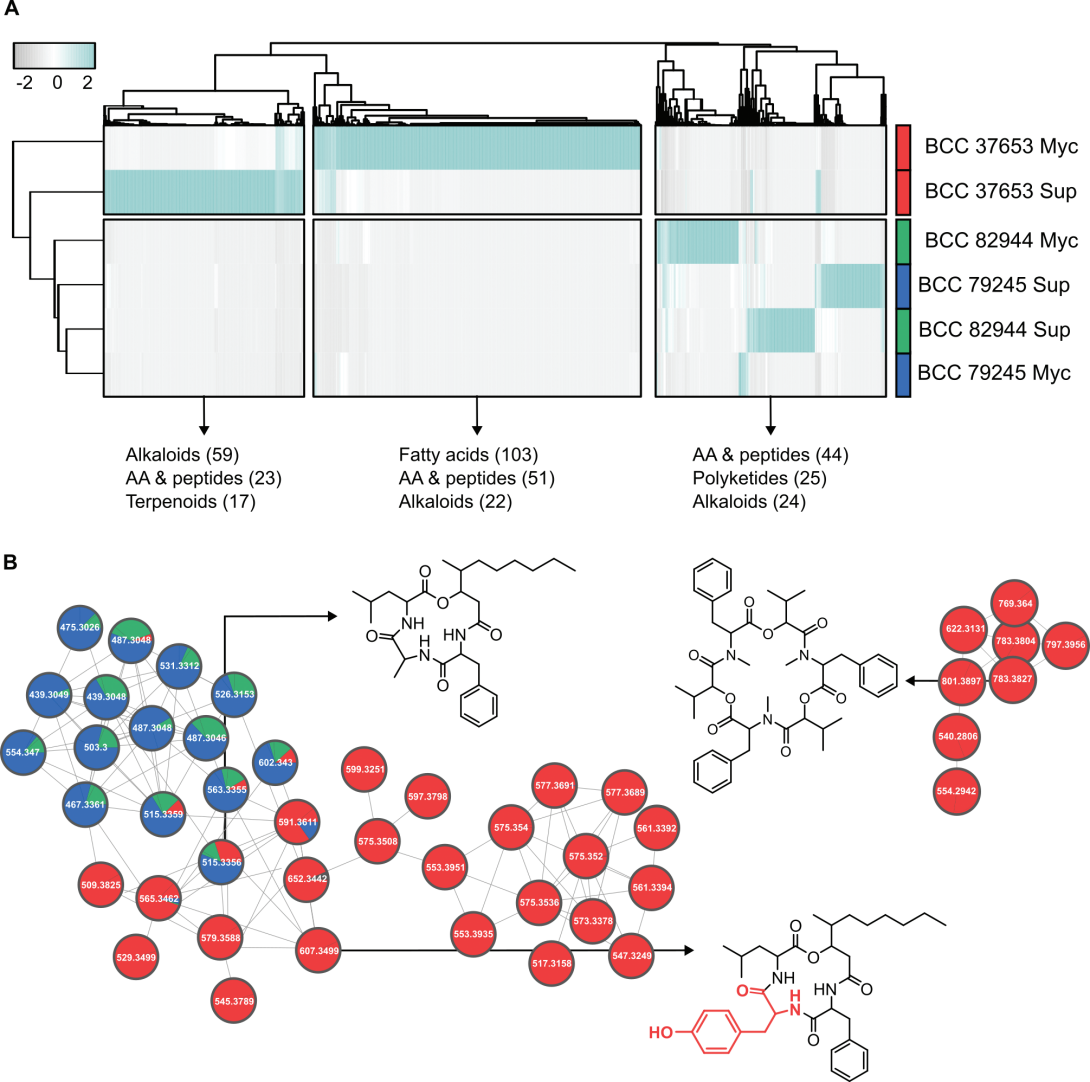
**A** Heatmap displaying the hierarchical clustering of features detected from the cultivation of *C. blackwelliae*BCC 37653 and *C. javanica* (BCC 79245 and BCC 82944) in YM liquid medium. The relative abundance of features within the crude extracts is visualized, and the three most abundant natural product classes, as predicted by CANOPUS, are highlighted for the major clusters. The heatmap, along with dendrograms, were generated using the R package pheatmap. **B** Molecular families related to beauveriolides and beauvericins were constructed using FBMN analysis of the features detected after cultivation in YM. Nodes are colored based on their relative production by each *Cordyceps* species. Structures shown are representative annotations of beauveriolide L and beauvericin, as well as the putative identification of tyrosine-containing beauveriolides in *C. blackwelliae*BCC 37653, supported by ModiFinder.

To expand our understanding of the chemical space produced by both *Cordyceps* species, natural product classes were predicted *de novo* from MS/MS spectra using CANOPUS (Dührkop et al. 2021; Valencia-Revelo et al. 2025). Hierarchical clustering analysis integrating CANOPUS annotations revealed clear differences in the metabolome of *C. blackwelliae* and *C. javanica* (Fig. 2A). While alkaloid- and fatty-acid-like features were the most abundant in extracts from *C. blackwelliae*, compounds classified as amino acids and peptides, including beauveriolides, were the major metabolites in *C. javanica*. Beauveriolides I, J_b_, and L, annotated by comparison with authentic standards, were not detected in *C. blackwelliae*. Our feature-based molecular networking (FBMN) analysis revealed a broader diversity of beauveriolides beyond annotations in both species. *Cordyceps blackwelliae* produced distinct analogs that clustered together within the same molecular family (MF) as the annotated beauveriolides. This suggests that, although the metabolome of *C. blackwelliae* is more diversified than that of *C. javanica*, beauveriolides production is conserved between the two species but with distinct amino acid rearrangements. Manual inspection supported by ModiFinder (Shahneh et al. 2024), suggested the presence of tyrosine-containing analogs in *C. blackwelliae*, exemplified by two features with neutral masses of 579.3588 and 607.3499 Da, which likely denote the substitution of alanine with tyrosine in beauveriolides I and L, respectively. The detection of beauvericins exclusively in *C. blackwelliae* suggests a broader chemical arsenal than *C. javanica*, which intriguingly correlates with the distinct virulence traits observed. Beauveriolides, which have been mainly characterized in *Beauveria bassiana*, are known to play a role in the early pathogenesis (Kim et al. 2024; Apirajkamol et al. 2025). The conservation of these metabolites in both *Cordyceps* species supports their likely involvement during infection, whereas the broader metabolome differentiation possibly reflects species-specific virulence mechanisms or host specialization strategies, also considering the phylogenetic distance between both taxa.

### ﻿Targeted Isolation and Structure Elucidation of Compounds

**Figure 3.**
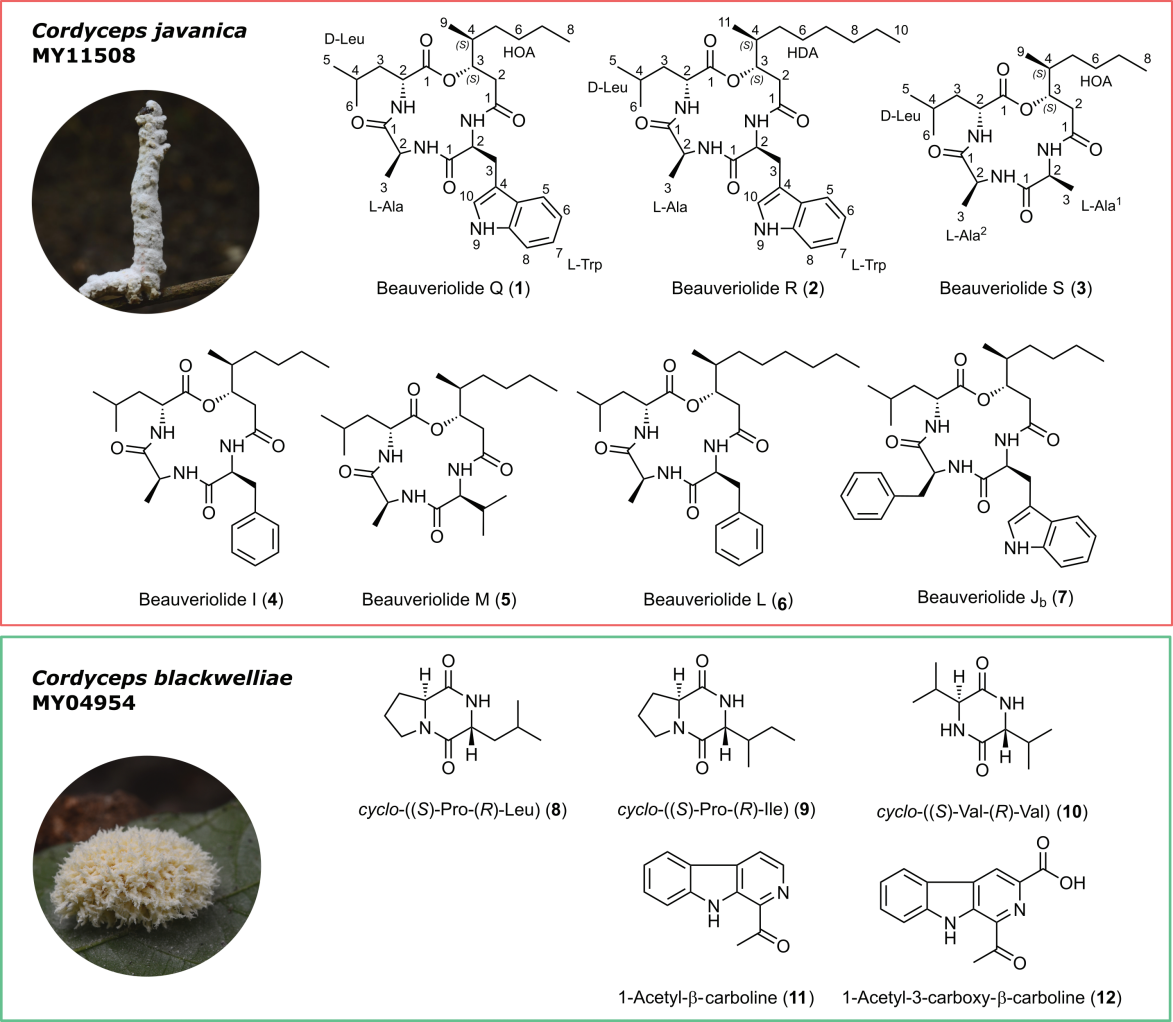
Chemical structures of the secondary metabolites isolated from *C. javanica*BCC 82944 (herbarium code MY11508) and *C. blackwelliae*BCC 37653 (herbarium code MY04954).

We hypothesize that beauveriolides are the authentic metabolites prevalent during infection, because diketopiperazines are broadly produced across various fungal lineages and are not exclusive to entomopathogens, making their role in infection less likely (Ma et al. 2016). Since both *C. javanica* strains produced beauveriolides in higher titers than *C. blackwelliae*, we systematically evaluated beauveriolide production in several media for BCC 82944 and BCC 79245. Notably, *C. javanica*BCC 82944 exhibited a broader diversity of beauveriolides, including features with previously unreported molecular formulas that suggested putatively novel congeners, and which could be obtained from the GG1 medium in higher yields. Although *C. blackwelliae* predominantly produced diketopiperazines *in vitro*, we scaled up its cultivation in YM medium to comprehensively profile its secondary metabolome. Chemical investigation of the crude extracts afforded seven beauveriolide congeners including three previously undescribed from *C. javanica*, while the crude extract of *C. blackwelliae* afforded three diketopiperazine and two *β*-carboline derivatives (Fig. 3).

**Figure 4.**
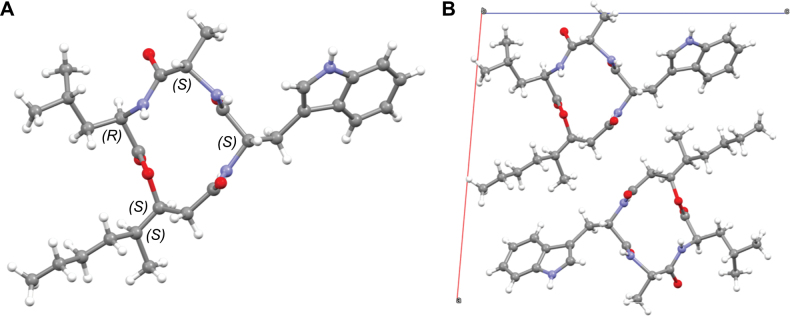
**A** Molecular conformation and absolute stereochemistry of beauveriolide Q (**1**) as determined from 3D ED. **B** Molecular packing within the crystal structure viewed along the b axis.

Compound **1** was obtained as a white powder, and its molecular formula was determined as C_29_H_42_N_4_O_5_ based on HR-ESI-MS data (Suppl. material 1: fig. S2), revealing a protonated molecular ion peak at *m/z* 527.3226 [M + H]^+^ and a sodium adduct at *m/z* 549.3046 [M + Na]^+^, indicating eleven degrees of unsaturation. Due to the poor solubility of **1** in deuterated solvents, NMR spectroscopy and attempts to grow suitable single crystals for X-ray diffraction were unsuccessful. Therefore, 3D electron diffraction (3D ED) was employed (Gemmi et al. 2019). Crystals of **1** had lateral dimensions in the micron range and were sufficiently thin to allow the collection of high-quality 3D ED data. Once the crystal structure was determined, we targeted dynamical refinement to determine its absolute stereochemistry (Klar et al. 2023). The 3D ED data workflow is complex, involving multiple steps of data conversion. During this process, pattern flipping can occur, potentially leading to an incorrect determination of the absolute structure. To address this, we pre-calibrated the entire data workflow using GRGDS, a 5-amino-acid peptide with a known stereochemistry (Suppl. material 1: fig. S3). Indeed, the workflow contained a pattern-flipping step, but once identified, this issue was resolved, enabling the reliable determination of the absolute stereochemistry of **1** (Fig. 4).

To our surprise, during our study and the preparation of this manuscript, a crystal structure and the absolute stereochemistry of beauveriolide I (**4**) was reported (Gurung et al. 2024), also based on 3D ED data. Interestingly, despite the different aromatic substituents and, consequently, distinct crystal structures, the macrocycle and aliphatic parts of the two molecules adopt nearly identical conformations. This suggests a relative rigidity of the macrocycle. An overlay of the two molecular conformations, **1** and beauveriolide I (**4**), is shown in Suppl. material 1: fig. S4.

Based on the obtained results, compound **1** was unambiguously reported as an undescribed beauveriolide featuring *cyclo*-(–C_9_–*L*-Trp–*L*-Ala–*D*-Leu) and named beauveriolide Q, whose molecular formula was tentatively reported in a chemotaxonomic study of *Beauveria* and *Paecilomyces* (Jegorov et al. 2004). It was mentioned as a synthetic chemical among many others in a Japanese patency as acyl-CoA:cholesterol acyltransferase 2 (ACAT-2) inhibitors with no spectral data included (Hiroshi et al. 2010).

Compound **2** was obtained as a white powder whose HR-ESI-MS (Suppl. material 1: fig. S7) established its molecular formula as C_31_H_46_N_4_O_5_ indicating eleven degrees of unsaturation similar to **1**. Compound **2** exhibited an additional C_2_H_4_ moiety when compared to **1**, as indicated by a mass difference of 28 Da. Unlike **1**, compound **2** was soluble in deuterated DMSO and therefore, its NMR spectral data (Table 1) could be obtained. The ^1^H NMR spectral data of **2** revealed the pattern of a peptide containing three N*H* proton signals (δ_H_ 7.19~8.42 ppm) and four *α*-protons at δ_H_ 3.90~4.39 ppm indicating the presence of three amino acid residues. In addition, the ^1^H NMR spectral data of **2** revealed an oxygenated multiplet proton at δ_H_ 4.84 (dt, *J* = 10.2, 5.3 Hz) that is directly correlated via the HSQC spectrum (Suppl. material 1: fig. S12) to an oxygenated sp^3^ carbon (δ_C_ 75.8).

**Figure 5.**
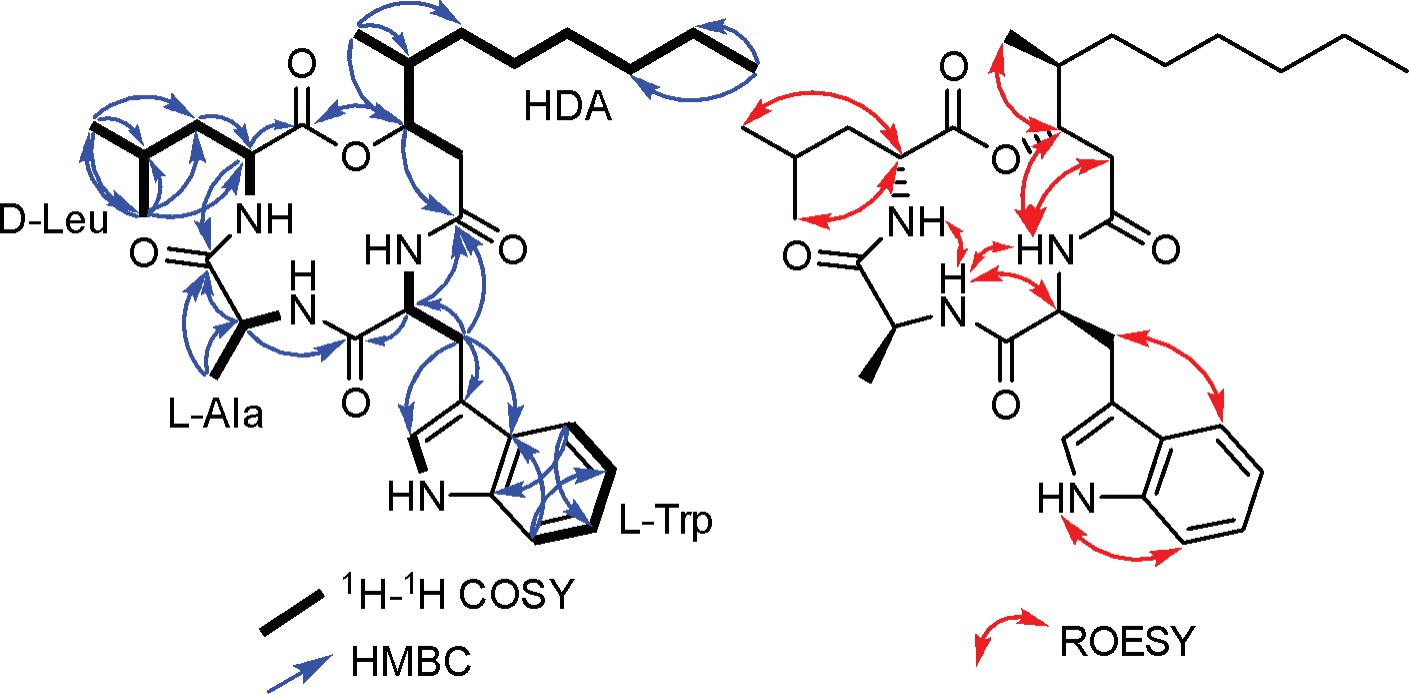
Key ^1^H–^1^H COSY, HMBC and ROESY correlations of **2**.

The ^1^H NMR and ^1^H–^1^H COSY spectral data of **2** (Fig. 5, Suppl. material 1: figs S8, S10) revealed the presence of a tryptophan residue (Trp) supported by the spin system extending among the characteristic four adjacent aromatic protons (δ_H_ 6.97~7.50 ppm) and a doublet aromatic proton at δ_H_ 7.11 (d, *J* = 2.5 Hz) correlated via ^1^H–^1^H COSY cross peak to an exchangeable N*H* proton at δ_H_ 10.86 (d, *J* = 2.5 Hz) of the indole ring.

A detailed investigation of ^1^H NMR and ^1^H–^1^H COSY spectra (Table 1, Fig. 5) suggested the presence of two amino acid residues namely; alanine (Ala) and leucine (Leu) supported by two spin systems as follows: 1) from an N*H* proton at δ_H_ 8.33 (d, *J* = 7.4 Hz) to an *α*-proton at δ_H_ 3.90 (p, *J* = 6.9 Hz) and a doublet methyl group at δ_H_ 1.12 (d, *J* = 6.9 Hz); 2) from an N*H* proton at δ_H_ 7.19 (d, *J* = 9.2 Hz) to an *α*-proton at δ_H_ 4.39 (q, *J* = 8.0 Hz), a triplet methylene group at δ_H_ 1.43 (t, *J* = 7.4 Hz) and a multiplet methine proton at δ_H_ 1.49 ending through two doublet methyl groups at δ_H_ 0.85/0.87 (d, *J* = 6.5 Hz). Therefore, the identity of **2** as beauveriolide R, previously reported only by tandem MS in a chemotaxonomic study of related genera, was confirmed. However, no detailed spectral data beyond MS/MS were available (Jegorov et al. 2004). From our NMR data, and in addition to the three amino acid residues, Trp–Ala–Leu, the fourth moiety was deduced to be 3-hydroxy-4-methyldecanoic acid (HDA) in agreement with the molecular formula. Both HMBC and ROESY correlations (Fig. 5, Suppl. material 1: figs S11, S13) established its amino acid sequence as *cyclo*-(–C_11_–Trp–Ala–Leu). Attempts to assign the absolute configurations using Marefy’s method were unsuccessful despite trying it twice. However, based on their common biosynthetic origin, supported by previous literature, and the available crystal structure of **1** from this study, the absolute configuration of **2** was assigned as *cyclo*-(–C_11_–*L*-Trp–*L*-Ala–*D*-Leu), consistent with other beauveriolides. Based on the obtained NMR spectral data, compound **2** was elucidated as a previously undescribed cyclodepsipeptide, beauveriolide R.

Compound **3** was obtained as a colorless oil. HR-ESI-MS (Suppl. material 1: fig. S15) established its molecular formula as C_21_H_37_N_3_O_5_, with [M + H]^+^ at *m/z* 412.2809 and [M + Na]^+^ at *m/z* 434.2625, indicating five degrees of unsaturation. Similar to **1**, this metabolite was insoluble in deuterated solvents and due to its oily nature, all crystallographic approaches, including 3D electron diffraction, were unsuccessful. These cyclodepsipeptides featuring one 3-hydroxy-4-methyl fatty acid, two *L*-amino acids, and one *D*-amino acid, exhibit characteristic MS/MS fragmentation patterns. Consequently, we conducted an in-depth MS/MS analysis of **3**. In FBMN and MS/MS comparison, compound **3** clustered together with beauveriolide I (**4**), which has a molecular mass of 487.3043 Da and comprises Phe, Ala, Leu, and a 3-hydroxy-4-methyloctanoyl residue (HOA). The 76 Da mass difference between the two compounds corresponds to the loss of a C_6_H_4_ group, which indicates the substitution of *L*-Phe with *L*-Ala in **3** (Suppl. material 1: fig. S5). Compound **3** also clustered with beauveriolide M (**5**), which has a molecular mass of 439.3048 Da and features *L*-Val instead of *L*-Phe. Both compounds generated common fragment ions at *m/z* 341.24, 281.19, 228.16, 210.15, 156.14, 139.12, and 132.11 Da, while the 28 Da difference between the two metabolites, attributed to a C_2_H_4_ group, indicates the substitution of *L*-Val with *L*-Ala in **3** (Suppl. material 1: fig. S5). Altogether, this evidence supports the identification of **3** as a previously undescribed derivative that was given a trivial name beauveriolide S.

Three additional beauveriolides, M (**5**), L (**6**), and J_b_ (**7**), were identified by a comparison of their HR-ESI-MS and NMR data with the previously reported literature (Jegorov et al. 1994; Kuzma et al. 2001; Jegorov et al. 2004; Helaly et al. 2019). In addition, chemical investigation of the crude extract derived from *C. blackwelliae*BCC 37653 in YM medium afforded five known compounds. These were identified as diketopiperazines, consistent with our MS/MS-based analysis, and were elucidated as *cyclo*-((*S*)-Pro-(*R*)-Leu) (**8**), *cyclo*-((*S*)-Pro-(*R*)-Ile) (**9**) and *cyclo*-((*S*)-Val-(*R*)-Val) (**10**), 1-acetyl-*β*-carboline (**11**) and 1-acetyl-3-carboxy-*β*-carboline (**12**) by comparisons of their HR-ESI-MS and NMR spectral data with the reported literature (Faini et al. 1978; Naveen et al. 2016; Bérubé et al. 2017; Simon et al. 2019; Shi et al. 2023).

### ﻿Biological properties of secondary metabolites and ecological relevance

All isolated compounds were subjected to antimicrobial assays against Gram-positive and Gram-negative bacteria, filamentous fungi, and yeasts. The obtained results (Suppl. material 1: table S3) revealed that beauveriolides S (**3**) and I (**4**) exhibited weak activity against *Staphylococcus aureus* (MIC = 66.6 µg/mL), with **4** also inhibiting *Bacillus subtilis* at the same concentration. Beauveriolide M (**5**) exhibited weak activity against *Mucor hiemalis*. All compounds were evaluated for their cytotoxic effects against two mammalian cell lines, with those showing strong activity subjected to further testing against five additional cell lines. Beauveriolides I (**4**) and J_b_ (**7**) (Suppl. material 1: table S4) displayed cytotoxic effects against human endocervical adenocarcinoma (KB3.1), with IC_50_ values of 13.3 and 36.5 µM, respectively. Compound **4**, however, was further evaluated against other cell lines, displaying potent cytotoxicity against human lung carcinoma (A549), human epidermoid carcinoma (A431), and human prostate carcinoma (PC-3) at IC_50_ values of 0.9, 1.2 and 3.5 µM, respectively. While for the compounds from *C. blackwelliae*, no antimicrobial or cytotoxic effects were observed.

Our findings suggest that beauveriolides, while not broadly antimicrobial, may play a more specialized role in *Cordyceps* entomopathogenic lifestyle. Their activity against specific microbes might reflect a dual role, modulating competitors within the insect host and weakening host defenses by targeting the microbiome, as seen in the case of helvolic acid (Sun et al. 2022). Furthermore, the cytotoxicity of certain beauveriolides might point towards potential direct toxicity to insect tissues. Although metabolites from *C. blackwelliae* showed no biological activity in our assays, FBMN underscored previously undescribed beauveriolides in its extracts. Unfortunately, these compounds could not be isolated in sufficient purity or quantity for structural or biological characterization, leaving room for future studies.

**Figure 6.**
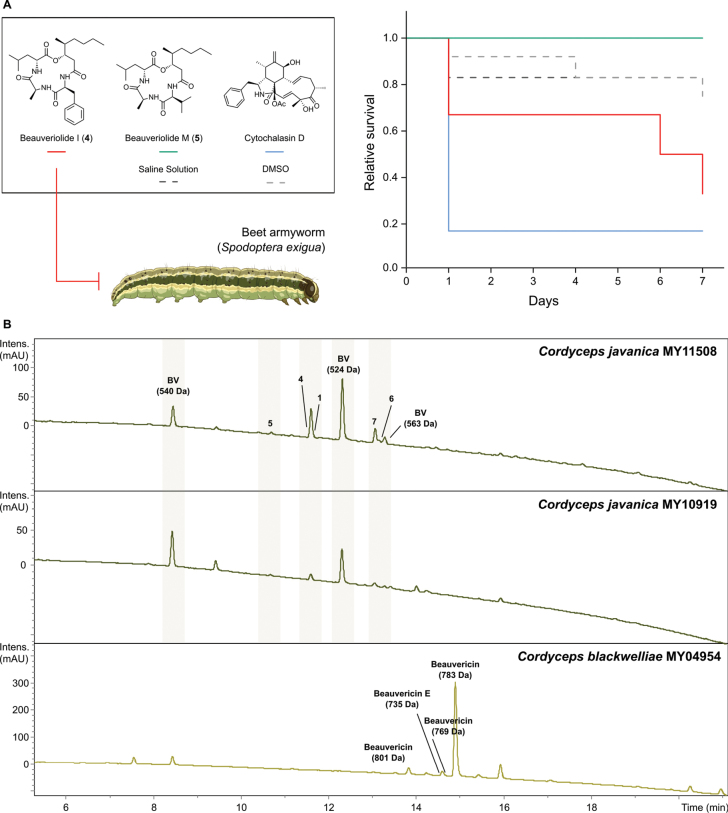
**A** Evaluation of the virulence of beauveriolides I (**4**) and M (**5**) produced by *C. javanica* against the beet armyworm (*fungiSpodoptera exigua*, Lepidoptera) over a 7-day period, considering the relative survival rate based on total mortality (TM). Cytochalasin D served as the positive control, while saline solution (8.5% NaCl) and DMSO were used as negative controls. **B** HPLC-UV/Vis chromatograms (210 nm) of the crude extracts obtained from the insect host corpses from *C. javanica* strains MY11508 and MY10919, and *C. blackwelliae* strain MY04954 herbarium material. Isolated metabolites are indicated by bold compound numbers. Putative beauveriolide derivatives are labeled as BV followed by their corresponding molecular weight. Similarly, detected beauvericins are labeled each with their name and molecular weight.

Given the modest cytotoxic and antimicrobial activity observed, we evaluated the virulence of purified beauveriolides against beet armyworm larvae. For proof of concept, beauveriolides I (**4**) and M (**5**) were selected based on availability and tested following the spore-suspension protocol but considering only total mortality, with cytochalasin D, a mycotoxin commonly produced by *Metarhizium* spp., used as a positive control (Kobmoo et al. 2024). Beauveriolide I (**4**) caused notable insect mortality with an average TM of 33% over the first five days and increasing to 67% TM in the last two days (Fig. 6). In contrast, beauveriolide M (**5**) showed no insecticidal over 7 days and resulted in lower TM than the two negative controls. Meanwhile, cytochalasin D caused rapid mortality, achieving 83% TM by day 2 (Fig. 6A). These results identify beauveriolide I (**4**) as a candidate effector in *Cordyceps* entomopathogenicity, because of its insecticidal activity and cytotoxicity against carcinoma cell lines, supporting its potential role in host colonization.

Our study highlights how amino acid substitutions modulate beauveriolides’ bioactivities. Replacement of the *L*-Trp residue in **1** with *L*-Phe, *L*-Ala, or *L*-Val resulted in distinct cytotoxicity and virulence profiles. Thus, the amino acid at this position is crucial for bioactivity. The lack of antimicrobial activity in some beauveriolides, such as those containing HDA, further supports the hypothesis that these molecules may have different functions, such as facilitating insect pathogenicity rather than broad antimicrobial antagonism. Beauveriolide I (**4**) showed insecticidal effects and strong cytotoxicity against carcinoma cell lines, with no apparent toxicity toward mouse fibroblast cells. Beauveriolides I and III have also been reported to exhibit potent antiatherogenic effects in macrophages without adverse outcomes in animal studies (Namatame et al. 2004). Additionally, beauveriolides have been detected during the late stages of silkworm larval infection by *B. bassiana* (Xu et al. 2015). Although the precise mechanisms underlying their selective *in vitro* activity remain uncertain, our findings support a generally non-toxic profile for the metabolites produced by both *Cordyceps* spp. representing a valuable step toward evaluating their potential use as biocontrol agents.

Finally, we examined the original insect cadavers from which both *Cordyceps* strains were isolated. Organic extraction of the herbarium material revealed the presence of beauveriolides in *C. javanica* and beauvericins in *C. blackwelliae* (Fig. 6B). This provides, to our knowledge, the first direct evidence that these metabolites are produced *in vivo* and confirms their role during host colonization. The distinct metabolomes revealed that *C. javanica* produced beauveriolides both *in vitro* and in cadavers, while *C. blackwelliae* produced diketopiperazines *in vitro* but beauvericins in cadavers, suggest species-specific metabolic programs and divergent ecological and/or host-adaptation strategies. Whether this pattern is an isolated case or widely distributed at the genus or family levels remains an open question. We believe that future studies should address the spatial and temporal action of these molecules during infection building upon concurrent work by Tehan et al. (Tehan et al. 2025), as well as their molecular targets and regulatory mechanisms. Altogether, our work highlights the potential of *Cordyceps* species as rich sources of structurally and functionally diverse secondary metabolites, advancing our understanding of fungal entomopathogenicity and informing the development of more effective and ecologically sound biocontrol agents.

## ﻿Conclusion

Our integrated comparative metabolomics and virulence analyses revealed that *Cordyceps* species employ distinct chemical strategies during insect infection. *Cordyceps javanica* consistently produced diverse beauveriolides both in axenic cultures and inside infected cadavers, whereas *C. blackwelliae* shifted from producing diketopiperazines *in vitro* to accumulating beauvericins in host tissue. The detection of these molecules in authentic insect corpses provides direct in-host chemical evidence that secondary metabolites are produced during natural infection, and bioassays with purified compounds showed that insecticidal activity is highly structure-dependent, with single amino-acid substitutions switching beauveriolides from inactive to lethal. Together, these results support a model in which metabolic reprogramming, together with structural diversification of cyclodepsipeptides, triggers species-specific virulence strategies. This chemical plasticity has practical implications for biocontrol development, identifying active effector molecules and safer biocontrol agents, as well as advancing our understanding of host specificity and fungal evolution. Moving forward, broader taxonomic sampling and integration of genomic/transcriptomic data, together with spatially resolved metabolite mapping, will be essential to link biosynthetic regulation to ecological function and to translate these insights into ecologically informed biocontrol solutions.

Last but not least, the current study also points toward another interesting phenomenon that goes back to the use of “cordicipitoid” ascomycetes like *Cordyceps* and *Ophiocordyceps* in Traditional Asian Medicine, where the “herbal” drugs traditionally used consisted of both, the fungal stroma and the insect that the fungus had colonized. From reports in the literature, we can be fairly sure that the metabolite profiles of stromata are very different from those encountered in the cultures. Once we know better what the insect carcasses contain, it may be possible to interpret the data on the efficacy. For instance, stromata of *Ophiocordyceps sinensis* and other species are sold on the market even as OTC drugs, but they are mainly known to contain cordycepin as an active principle (cf. Liu et al. 2015). The mycelial cultures of these fungi are known to contain different major metabolites, but the current study is the first to investigate the host carcasses that are also supposed to be part of the TCM drugs (see discussion in Paterson 2008; Kinjo and Zang 2001).

## ﻿Additional information

### Use of AI

No use of AI was reported.

### Adherence to national and international regulations

All the fungal strains used in this study have been legally obtained, respecting the Convention on Biological Diversity (Rio Convention).

### Author ORCIDs

Esteban Charria-Girón https://orcid.org/0000-0002-3823-1416

Rita Toshe https://orcid.org/0009-0005-9068-5430

Artit Khonsanit https://orcid.org/0009-0009-0931-4288

Noppol Kobmoo https://orcid.org/0000-0002-3261-9970

Tatiana E. Gorelik https://orcid.org/0000-0002-0911-2039

Janet Jennifer Luangsa-ard https://orcid.org/0000-0001-6801-2145

Sherif S. Ebada https://orcid.org/0000-0002-2753-0031

Marc Stadler https://orcid.org/0000-0002-7284-8671

### Data availability

All of the data that support the findings of this study are available in the main text or Supplementary Information.

## Supplementary materials

Supplementary material 1 Additional information Data type docx https://binary.pensoft.net/file/1490659This dataset is made available under the Open Database License (http://opendatacommons.org/licenses/odbl/1.0/). The Open Database License (ODbL) is a license agreement intended to allow users to freely share, modify, and use this Dataset while maintaining this same freedom for others, provided that the original source and author(s) are credited.Esteban Charria-Girón, Rita Toshe, Artit Khonsanit, Noppol Kobmoo, Papichaya Kwanthong, Tatiana E. Gorelik, Janet Jennifer Luangsa-ard, Sherif S. Ebada, Marc Stadler
