# Supplementary material for: ﻿Chemical clues to infection: A pilot study on the differential secondary metabolite production during the life cycle of selected Cordyceps species
Source: IMA Fungus. 2025 Dec 12;16:e172651. doi: 10.3897/imafungus.16.172651 (PMC12717519; doi:10.3897/imafungus.16.172651)
Supplement: Supplementary material 1 — Additional information [file imafungus-16-e172651-s001.docx]

Supporting Information for:

**Chemical clues to infection: Metabolome differentiation underlies host colonization by biocontrol agents of the genus *Cordyceps***

**Short title:** *Cordyceps* chemical traits of infection.

Esteban Charria-Girón,[^1,^](#_heading=h.94wrwsp7sak9)^2,^[^†^](#_heading=h.94wrwsp7sak9)* Rita Toshe,^1^[^†^](#_heading=h.94wrwsp7sak9) Artit Khonsanit,^3^ Noppol Kobmoo,^4^ Papichaya Kwanthong,^4^ Tatiana E. Gorelik,^5,6,7^ Janet Jennifer Luangsa-ard,^4^ Sherif S. Ebada, ^1,8^* and Marc Stadler,[^1,^](#_heading=h.94wrwsp7sak9)^9^*

^1^ Department Microbial Drugs, Helmholtz Centre for Infection Research, Inhoffenstraße 7, 38124 Braunschweig, Germany.

^2^ Bioinformatics Group, Wageningen University & Research, Droevendaalsesteeg 1, 6708 PB Wageningen, the Netherlands.

^3^ 67 Moo 9 Ban Huai, Muang Nong Hang, Kuchinarai, Kalasin, Thailand.

^4^ Plant Microbe Interaction Research Team, BIOTEC, National Science and Technology Development Agency (NSTDA) 111 Thailand Science Park, Phahonyothin Road, Khlong Nueng, Khlong Luang, Pathum Thani, 12120, Thailand.

^5^ Department Structure and Function of Proteins, Helmholtz Centre for Infection Research (HZI), 38124 Braunschweig, Germany.

^6^ Department Microbial Natural Products, Helmholtz-Institute for Pharmaceutical Research Saarland (HIPS), 66123 Saarbrücken, Germany.

^7^ Ernst Ruska-Centre for Microscopy and Spectroscopy with Electrons (ER-C), Forschungszentrum Jülich, 52425 Jülich, Germany.

^8^ Department of Pharmacognosy, Faculty of Pharmacy, Ain Shams University, 11566 Cairo, Egypt.

^9^ Institute of Microbiology, Technische Universität Braunschweig, Spielmannstraße 7, 38106 Braunschweig, Germany.

**Author Notes:**

[^†^](#_heading=h.94wrwsp7sak9) Co-first authorship

*Corresponding author

Contents of Supporting Information

| # | Contents | Page |
| --- | --- | --- |
| 1 | Figure S1. LR-ESI-MS spectrum of **1**. | S4 |
| 2 | Figure S2. HR-ESI-MS spectrum of **1**. | S5 |
| 3 | Table S1. Crystallographic data for **GRGDS TFA** and **Beauveriolide Q** | S6 |
| 4 | Table S2. Dynamical refinement of **GRGDS TFA** and **Beauveriolide Q** | S7 |
| 5 | Figure S3. Absolute structure determination of **GRGDS TFA** – calibration of the data workflow. Left: the model as obtained in the structure determination procedure, right: the inverted model, bottom: the expected stereochemistry of **GRGDS**. | S8 |
| 6 | Figure S4. Overlay of the molecular conformations of compound **1** (stick representation) and beauveriolide I (**4**) (CSD code 2332378, wire representation) as determined from electron diffraction data. | S8 |
| 7 | Figure S5. Molecular family encompassing beauveriolides produced by *Cordyceps javanica* BCC 82944. Hexagon-shaped nodes represent isolated congeners, while figure-eight–shaped nodes indicate dereplicated beauveriolides with putative annotations based on previously reported structures. Suggested structural additions or losses between nodes are highlighted for the isolated metabolites. | S9 |
| 8 | Figure S6. LR-ESI-MS spectrum of **2**. | S10 |
| 9 | Figure S7. HR-ESI-MS spectrum of **2**. | S11 |
| 10 | Figure S8. ^1^H NMR spectrum of **2** in DMSO-*d*_6_ at 500 MHz. | S12 |
| 11 | Figure S9 ^13^C NMR spectrum of **2** in DMSO-*d*_6_ at 125 MHz. | S13 |
| 12 | Figure S10. ^1^H–^1^H COSY spectrum of **2** in DMSO-*d*_6_ at 500 MHz. | S14 |
| 13 | Figure S11. HMBC spectrum of **2** in DMSO-*d*_6_ at 500 MHz. | S15 |
| 14 | Figure S12. HSQC spectrum of **2** in DMSO-*d*_6_ at 500 MHz. | S16 |
| 15 | Figure S13. ROESY spectrum of **2** in DMSO-*d*_6_ at 500 MHz. | S17 |
| 16 | Figure S14. LR-ESI-MS spectrum of **3**. | S18 |
| 17 | Figure S15. HR-ESI-MS spectrum of **3**. | S19 |
| 18 | Figure S16. LR-ESI-MS spectrum of **4**. | S20 |
| 19 | Figure S17. HR-ESI-MS spectrum of **4**. | S21 |
| 20 | Figure S18. ^1^H NMR spectrum of **4** in DMSO-*d*_6_ at 500 MHz. | S22 |
| 21 | Figure S19. ^1^H–^1^H COSY spectrum of **4** in DMSO-*d*_6_ at 500 MHz. | S23 |
| 22 | Figure S20. LR-ESI-MS spectrum of **5**. | S24 |
| 23 | Figure S21. ^1^H NMR spectrum of **5** in DMSO-*d*_6_ at 500 MHz. | S25 |
| 24 | Figure S22. ^1^H–^1^H COSY spectrum of **5** in DMSO-*d*_6_ at 500 MHz. | S26 |
| 25 | Figure S23. LR-ESI-MS spectrum of **6**. | S27 |
| 26 | Figure S24. HR-ESI-MS spectrum of **6**. | S28 |
| 27 | Figure S25. ^1^H NMR spectrum of **6** in DMSO-*d*_6_ at 500 MHz. | S29 |
| 28 | Figure S26. LR-ESI-MS spectrum of **7**. | S30 |
| 29 | Figure S27. HR-ESI-MS spectrum of **7**. | S31 |
| 30 | Figure S28. ^1^H NMR spectrum of **7** in DMSO-*d*_6_ at 500 MHz. | S32 |
| 31 | Figure S29. ^1^H–^1^H COSY spectrum of **7** in DMSO-*d*_6_ at 500 MHz. | S33 |
| 32 | Figure S30. HMBC spectrum of **7** in DMSO-*d*_6_ at 500 MHz. | S34 |
| 33 | Figure S31. HSQC spectrum of **7** in DMSO-*d*_6_ at 500 MHz. | S35 |
| 34 | Figure S32. LR-ESI-MS spectrum of **8**. | S36 |
| 35 | Figure S33. HR-ESI-MS spectrum of **8**. | S37 |
| 36 | Figure S34. ^1^H NMR spectrum of **8** in DMSO-*d*_6_ at 500 MHz. | S38 |
| 37 | Figure S35. LR-ESI-MS spectrum of **9**. | S39 |
| 38 | Figure S36. HR-ESI-MS spectrum of **9**. | S40 |
| 39 | Figure S37. ^1^H NMR spectrum of **9** in DMSO-*d*_6_ at 500 MHz. | S41 |
| 40 | Figure S38. ^13^C NMR spectrum of **9** in DMSO-*d*_6_ at 125 MHz. | S42 |
| 41 | Figure S39. ^1^H–^1^H COSY spectrum of **9** in DMSO-*d*_6_ at 500 MHz. | S43 |
| 42 | Figure S40. HMBC spectrum of **9** in DMSO-*d*_6_ at 500 MHz. | S44 |
| 43 | Figure S41. HSQC spectrum of **9** in DMSO-*d*_6_ at 500 MHz. | S45 |
| 44 | Figure S42. ROESY spectrum of **9** in DMSO-*d*_6_ at 500 MHz. | S46 |
| 45 | Figure S43. LR-ESI-MS spectrum of **10**. | S47 |
| 46 | Figure S44. HR-ESI-MS spectrum of **10**. | S48 |
| 47 | Figure S45. ^1^H NMR spectrum of **10** in DMSO-*d*_6_ at 500 MHz. | S49 |
| 48 | Figure S46. ^1^H–^1^H COSY spectrum of **10** in DMSO-*d*_6_ at 500 MHz. | S50 |
| 49 | Figure S47. HMBC spectrum of **10** in DMSO-*d*_6_ at 500 MHz. | S51 |
| 50 | Figure S48. ROESY spectrum of **10** in DMSO-*d*_6_ at 500 MHz. | S52 |
| 51 | Figure S49. LR-ESI-MS spectrum of **11**. | S53 |
| 52 | Figure S50. ^1^H NMR spectrum of **11** in DMSO-*d*_6_ at 500 MHz. | S54 |
| 53 | Figure S51. DEPTQ spectrum of **11** in DMSO-*d*_6_ at 125 MHz. | S55 |
| 54 | Figure S52. ^1^H–^1^H COSY spectrum of **11** in DMSO-*d*_6_ at 500 MHz. | S56 |
| 55 | Figure S53. HMBC spectrum of **11** in DMSO-*d*_6_ at 500 MHz. | S57 |
| 56 | Figure S54. HSQC spectrum of **11** in DMSO-*d*_6_ at 500 MHz. | S58 |
| 57 | Figure S55. ROESY spectrum of **11** in DMSO-*d*_6_ at 500 MHz. | S59 |
| 58 | Figure S56. HR-ESI-MS spectrum of **12**. | S60 |
| 59 | Figure S57. ^1^H NMR spectrum of **12** in DMSO-*d*_6_ at 500 MHz. | S61 |
| 60 | Figure S58. ^1^H–^1^H COSY spectrum of **12** in DMSO-*d*_6_ at 500 MHz. | S62 |
| 61 | Figure S59. HMBC spectrum of **12** in DMSO-*d*_6_ at 500 MHz. | S63 |
| 61 | Figure S60. HSQC spectrum of **12** in DMSO-*d*_6_ at 500 MHz. | S64 |
| 62 | Table S3. Antimicrobial properties of the isolated metabolites. | S65 |
| 63 | Table S4. Cytotoxic properties of the isolated metabolites. | S65 |


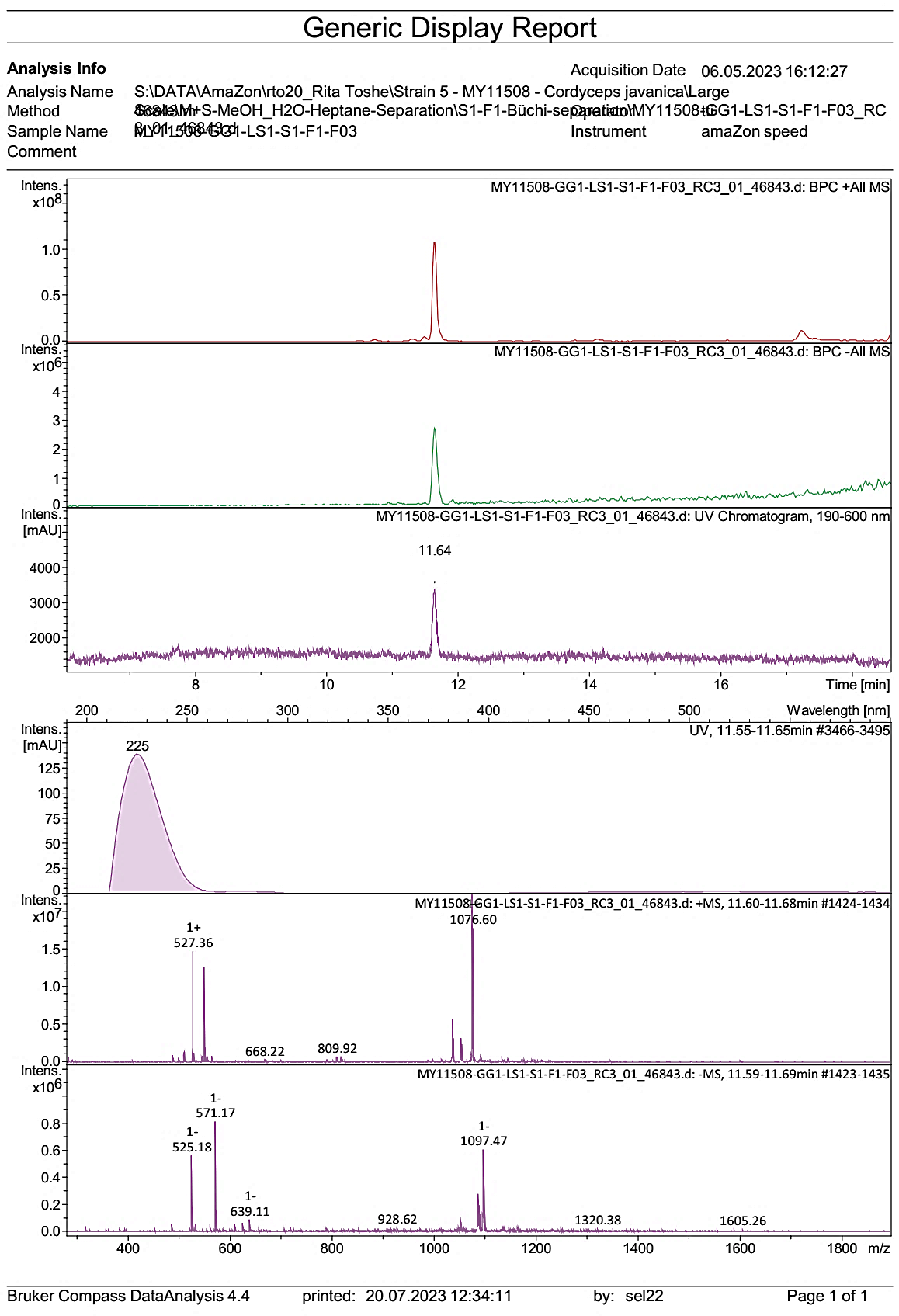


[2M+Na]^+^

[M+Na]^+^

**1+**

**549.35**

[M+H]^+^

[M-H]^-^

Figure S1. LR-ESI-MS spectrum of **1**.


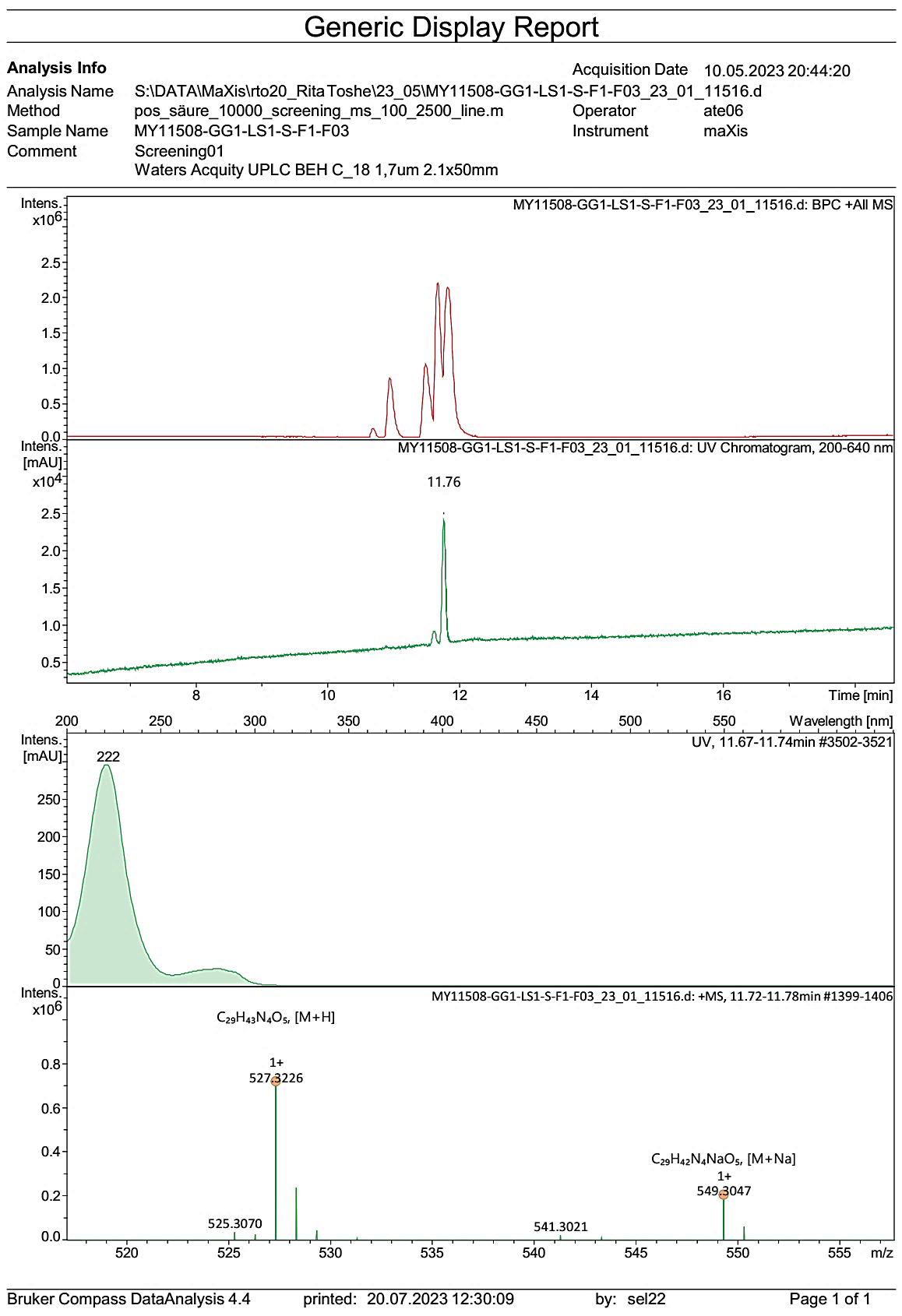


Figure S2. HR-ESI-MS spectrum of **1**.

**Structure determination of GRGDS, calibration of the data workflow for absolute structure determinaiton of Beauveriolide Q**

**GRGDS** is a five-amino-acid peptide used for the calibration of the absolute structure determination procedure with electron diffraction. The compound was purchased from GenScript Biotech (Leiden, Netherlands). Needle-like crystals, less than 0.5 µm wide and approximately 10 µm long, were grown from methanol. 3D ED data of **GRGDS** was collected using EPU-D and processed with PETS2, exactly as described for **Beauveriolide Q** in the Methods section of the main text.

The best-performing dataset was selected for structure analysis. The structure was solved with SHELXD and refined kinematically with SHELXL in the Olex2 environment. The crystallographic details are provided in Table S1.

Table S1. Crystallographic data for **GRGDS TFA** and **Beauveriolide Q**

|  | **GRGDS TFA** | **Beauveriolide Q** |
| --- | --- | --- |
| Crystal data |  | |
| Chemical formula | C_17_H_28_N_8_O_8_ F_3_O_2_C_2_H_1_ | C_29_H_42_N_4_O_5_ |
| Mr | 586.47 | 526.67 |
| Crystal system, space group | Monoclinic, *C2* | Monoclinic, *P2_1_* |
| Temperature (K) | 77 | 77 |
| a, b, c (Å) | 29.231, 4.546, 19.640 | 16.586, 5.170, 17.340 |
| β (°) | 106.70 | 94.91 |
| V (Å^3^) | 2499.8 | 1481.5 |
| Z, Z’ | 4, 1 | 2, 1 |
| Radiation type | electrons, λ = 0.0251 Å | electrons, λ = 0.0251 Å |
| Crystal size (mm) | 0.005 x 0.001 x 0.0002 | 0.008 x 0.001 x 0.0002 |
| Data collection |  | |
| Diffractometer | Thermo Fisher GLACIOS, EPU-D | Thermo Fisher GLACIOS, EPU-D |
| No. of measured, independent and observed [I > 2σ(I)] reflections | 4085, 2632, 2101 | 5306, 3513, 3221 |
| Rint | 0.131 | 0.137 |
| θmax (°) | 0.8 | 0.8 |
| (sin θ/λ)max (Å^−1^) | 0.550 | 0.569 |
| Data resolution, Å | 0.91 | 0.91 |
| Data completeness | 0.722 | 0.761 |
| Refinement |  | |
| R[F^2^ > 2σ(F^2^)], wR(F2), S | 0.173, 0.429, 1.64 | 0.256, 0.637, 3.31 |
| No. of reflections | 2632 | 3513 |
| No. of parameters | 178 | 146 |
| No. of restraints | 1 | 1 |
| H-atom treatment | H atoms treated by a mixture of independent and constrained refinement | H-atom parameters constrained |
| Absolute structure: dynamical refinement in JANA, R_obs_ | 15.62 / 12.50 | 20.79 / 22.09 |
| CSD deposition code | 2391399 | 2391744 |
| Original diffraction data | doi.org/10.5281/zenodo.13938423 | doi.org/10.5281/zenodo.13943915 |

Computer programs: *SHELXD* (Sheldrick, 2008), *SHELXL* 2018/3 (Sheldrick, 2015), Olex2 1.5 (Dolomanov *et al.*, 2009).

To our surprise, **GRGDS** co-crystallized with trifluoroacetic acid (**TFA**), which was evidently used in the peptide synthesis.

We then performed the absolute structure determination of the **GRGDS TFA** co-crystal through dynamical refinement from electron diffraction data using JANA. We did not carry out a full dynamical refinement of all structure parameters, focusing solely on the absolute structure determination.

The refinement statistics of the initial structure model obtained during structure determination showed worse figures of merit than the inverted enantiomer structure (Figure S3, Table S2).

Despite the higher R-factors obtained in the dynamical refinement (Table S2), the initial stereochemistry of the **GRGDS** molecule is correct. This suggests that somewhere in the data processing pipeline, a flipping of the diffraction patterns occurs. Instead of searching for the point where the data flipping occurs and correcting it, we now consider the entire procedure calibrated: the correct enantiomer should yield higher R-factors in the dynamical refinement.

Table S2. Dynamical refinement of **GRGDS TFA** and **Beauveriolide Q**

|  | R_obs_ | R_all_ | wR2_all_ |
| --- | --- | --- | --- |
| **GRGDS TFA** | 15.62 | 17.10 | 31.89 |
| **GRGDS TFA** inverted model | 12.50 | 13.98 | 24.50 |
| **Beauveriolide Q** | 20.79 | 22.19 | 40.44 |
| **Beauveriolide Q** inverted model | 22.09 | 23.46 | 42.80 |


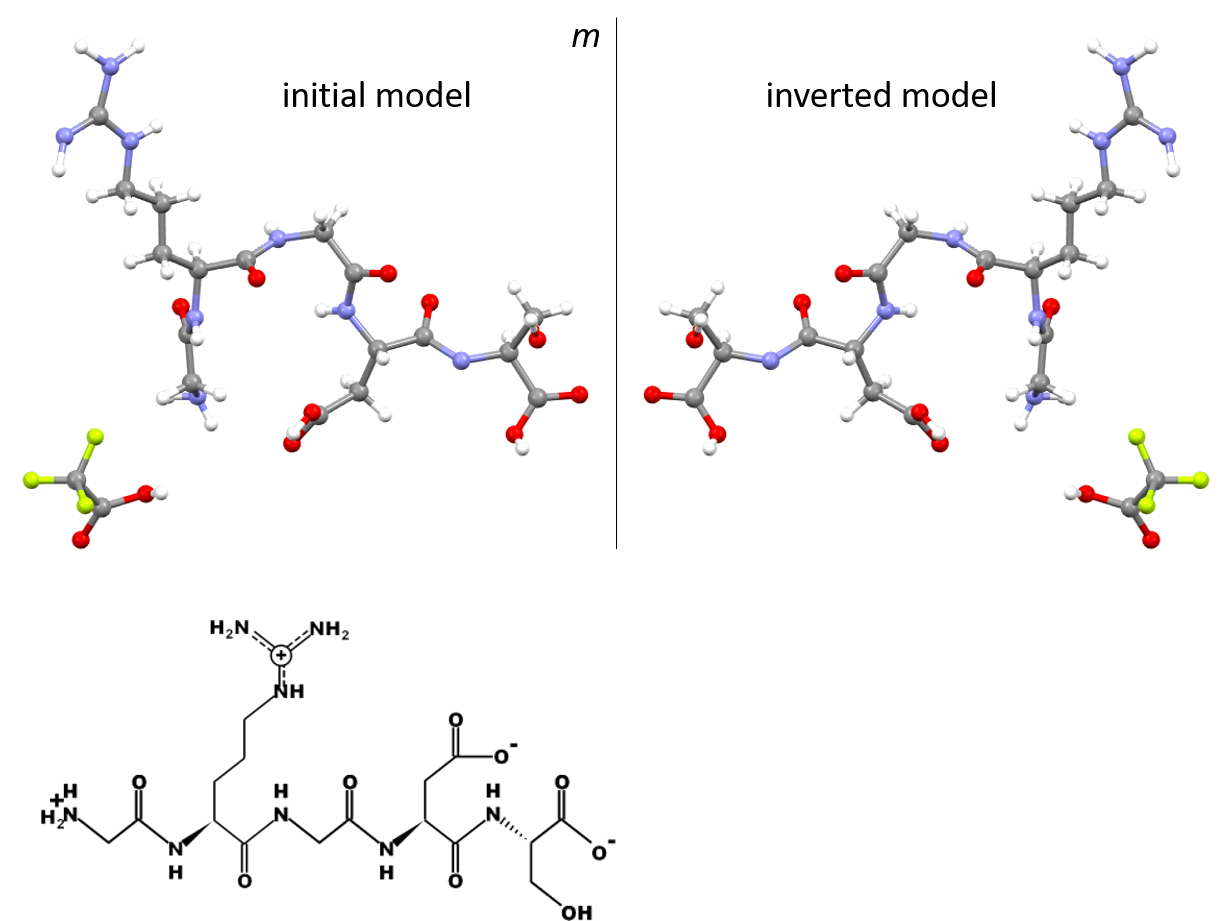


Figure S3. Absolute structure determination of **GRGDS TFA** – calibration of the data workflow. Left: the model as obtained in the structure determination procedure, right: the inverted model, bottom: the expected stereochemistry of **GRGDS**.

The same dynamical refinement procedure was then applied to **Beauveriolide Q**. The inverted model showed higher R-factors, indicating that it has the correct stereochemistry. The correct stereochemistry of MY and molecular packing within the crystal structure is illustrated in Figure S2.


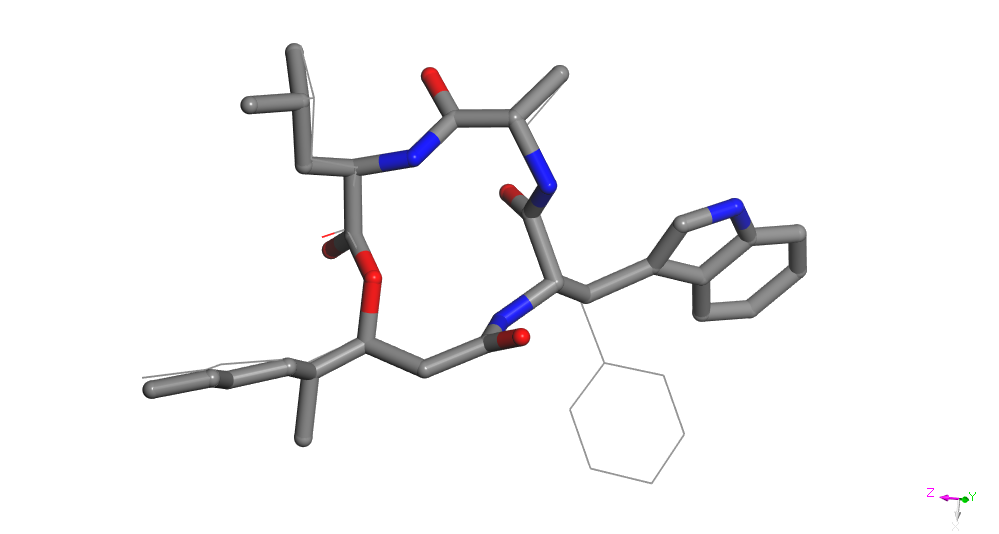


Figure S4. Overlay of the molecular conformations of compound **1** (stick representation) and beauveriolide I (**4**) (CSD code 2332378, wire representation) as determined from electron diffraction data.


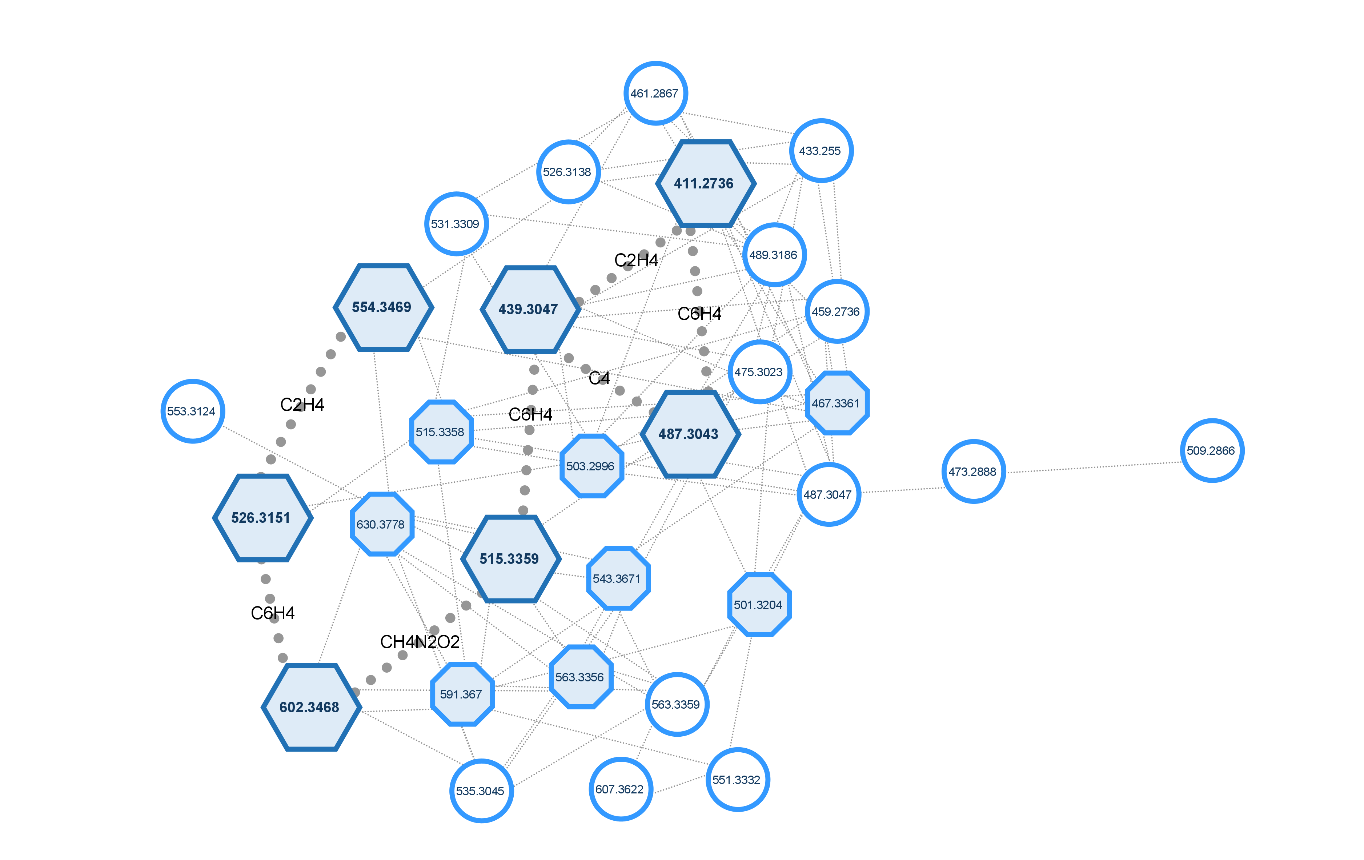


Figure S5. Molecular family encompassing beauveriolides produced by *Cordyceps javanica* BCC 82944. Hexagon-shaped nodes represent isolated congeners, while figure-eight–shaped nodes indicate dereplicated beauveriolides with putative annotations based on previously reported structures. Suggested structural additions or losses between nodes are highlighted for the isolated metabolites.


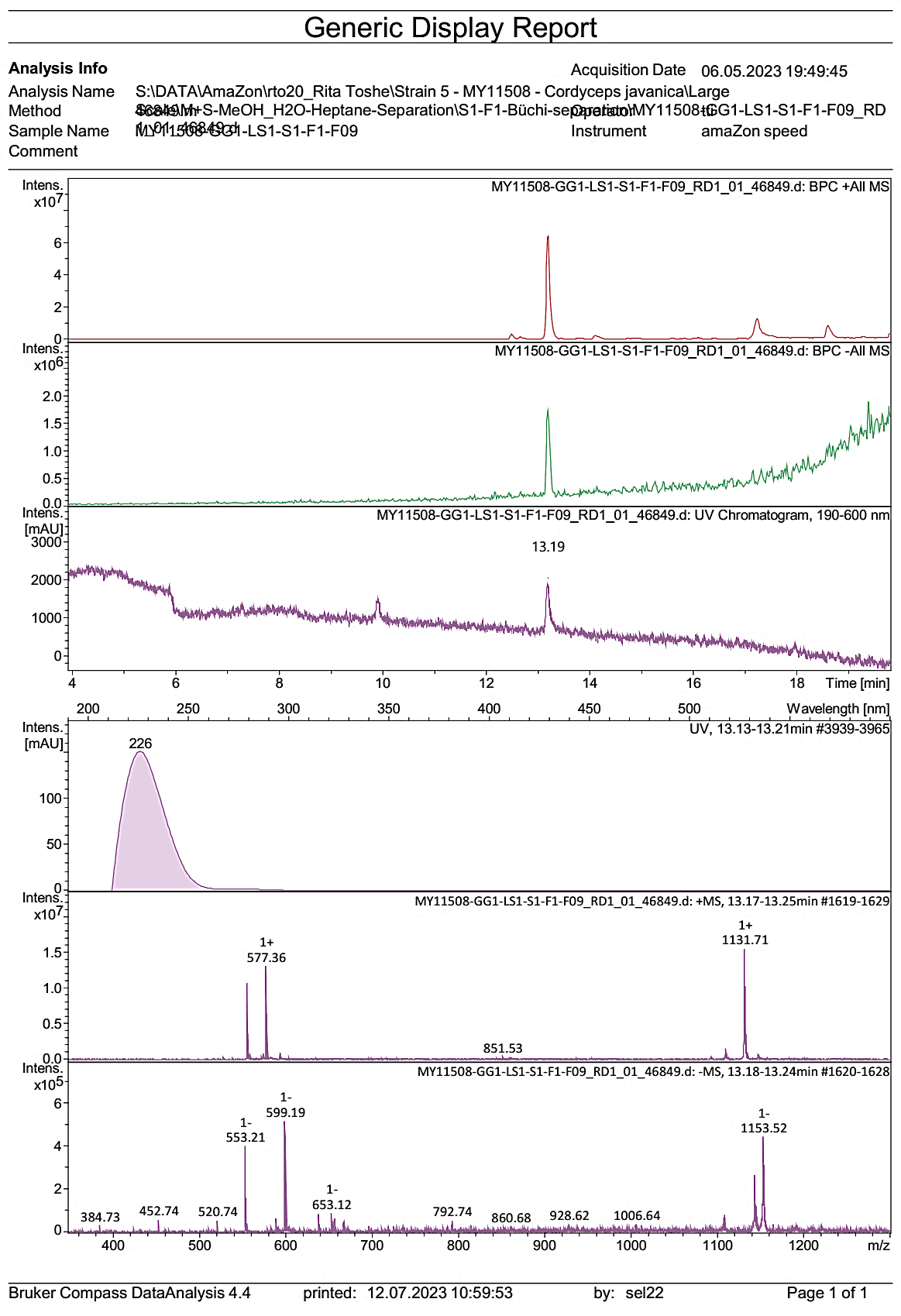


[2M+Na]^+^

[M-H]^-^

[M+H]^+^

[M+Na]^+^

**1+**

**555.41**

Figure S6. LR-ESI-MS spectrum of **2**.


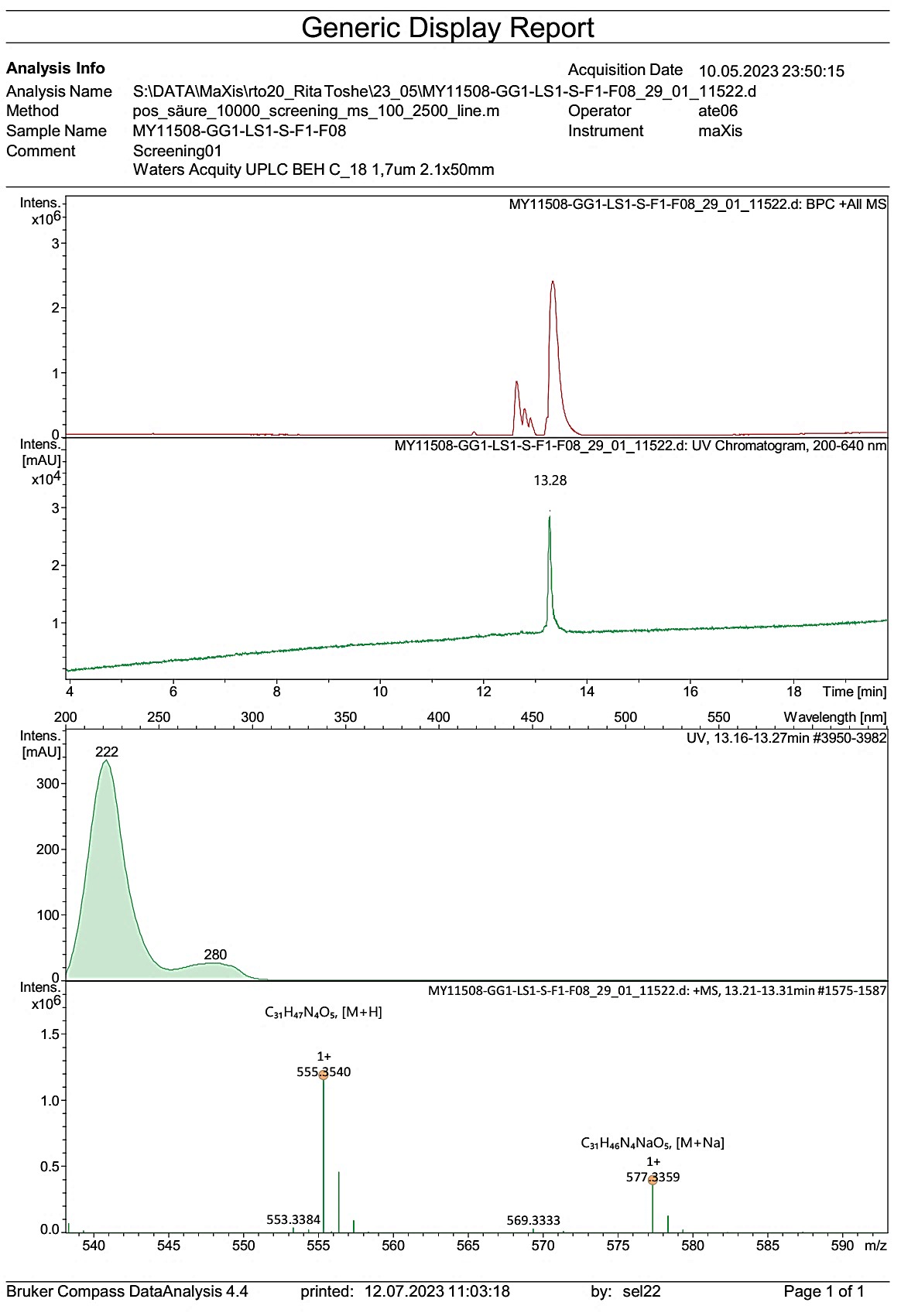


Figure S7. HR-ESI-MS spectrum of **2**.

Figure S8. ^1^H NMR spectrum of **2** in DMSO-*d*_6_ at 500 MHz.

Figure S9. ^13^C NMR spectrum of **2** in DMSO-*d*_6_ at 125 MHz.

Figure S10. ^1^H–^1^H COSY spectrum of **2** in DMSO-*d*_6_ at 500 MHz.

Figure S11. HMBC spectrum of **2** in DMSO-*d*_6_ at 500 MHz.

Figure S12. HSQC spectrum of **2** in DMSO-*d*_6_ at 500 MHz.

Figure S13. ROESY spectrum of **2** in DMSO-*d*_6_ at 500 MHz.


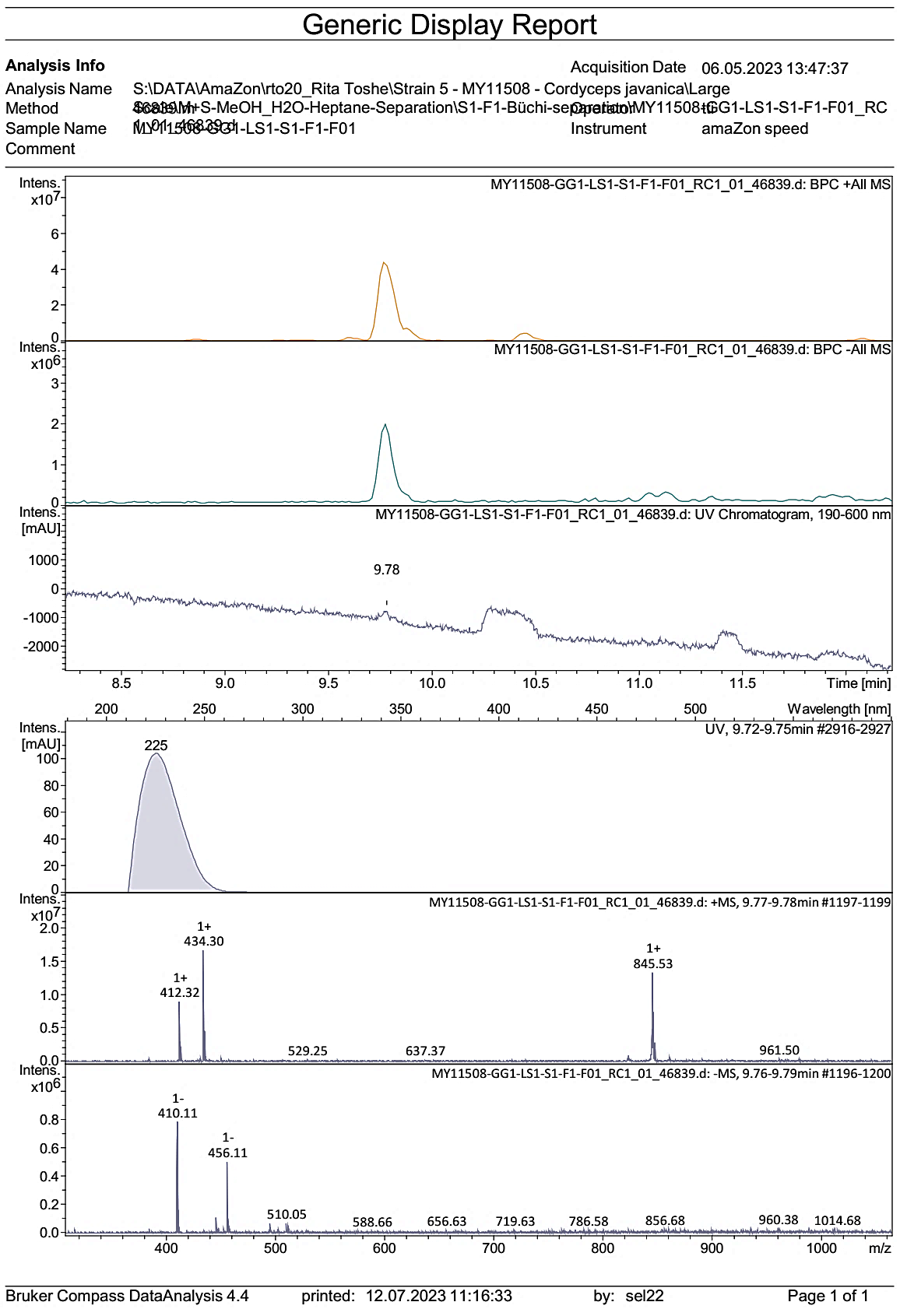


[2M+Na]^+^

[M+H]^+^

[M-H]^-^

[M+Na]^+^

Figure S14. LR-ESI-MS spectrum of **3**.


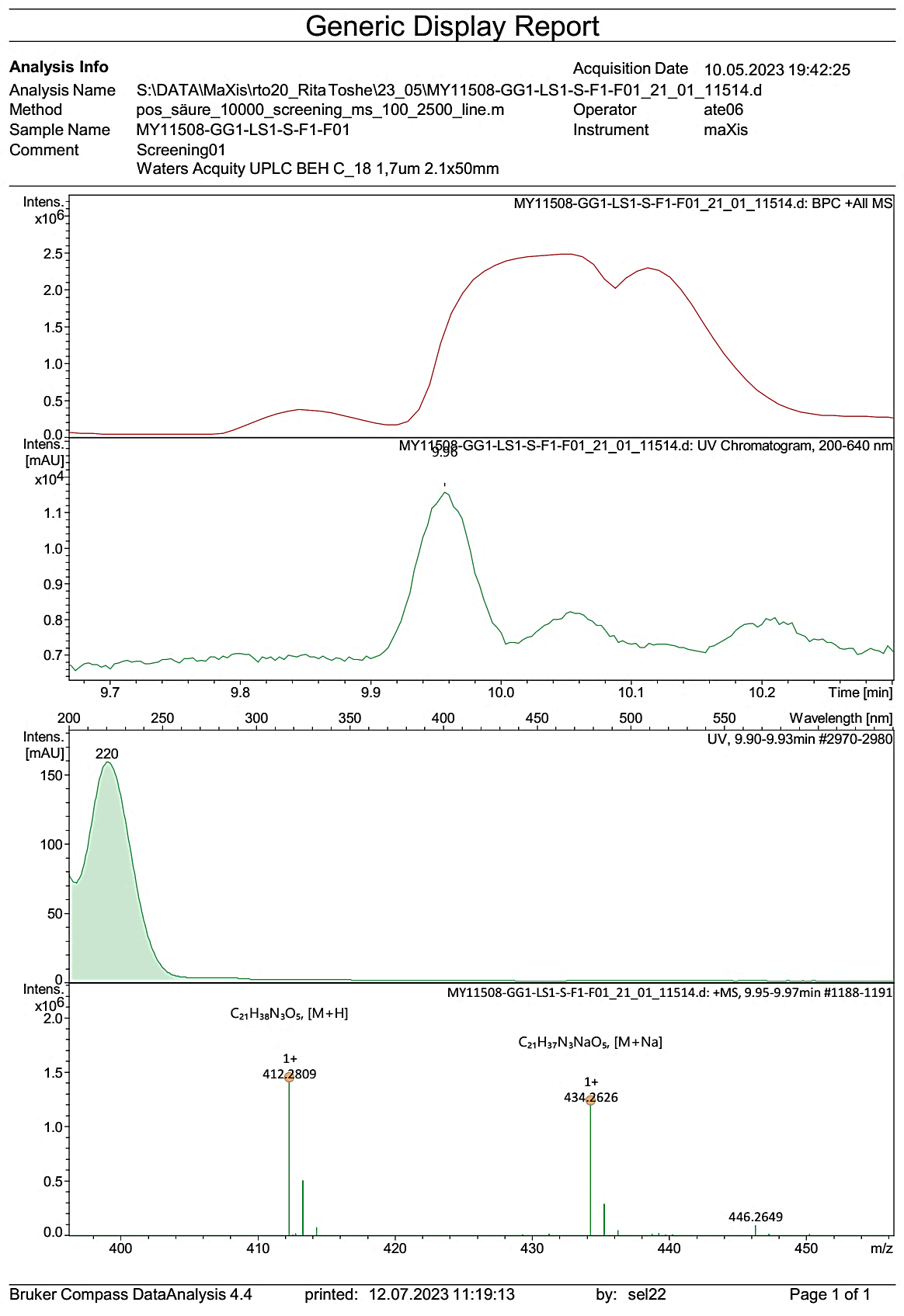


Figure S15. HR-ESI-MS spectrum of **3**.


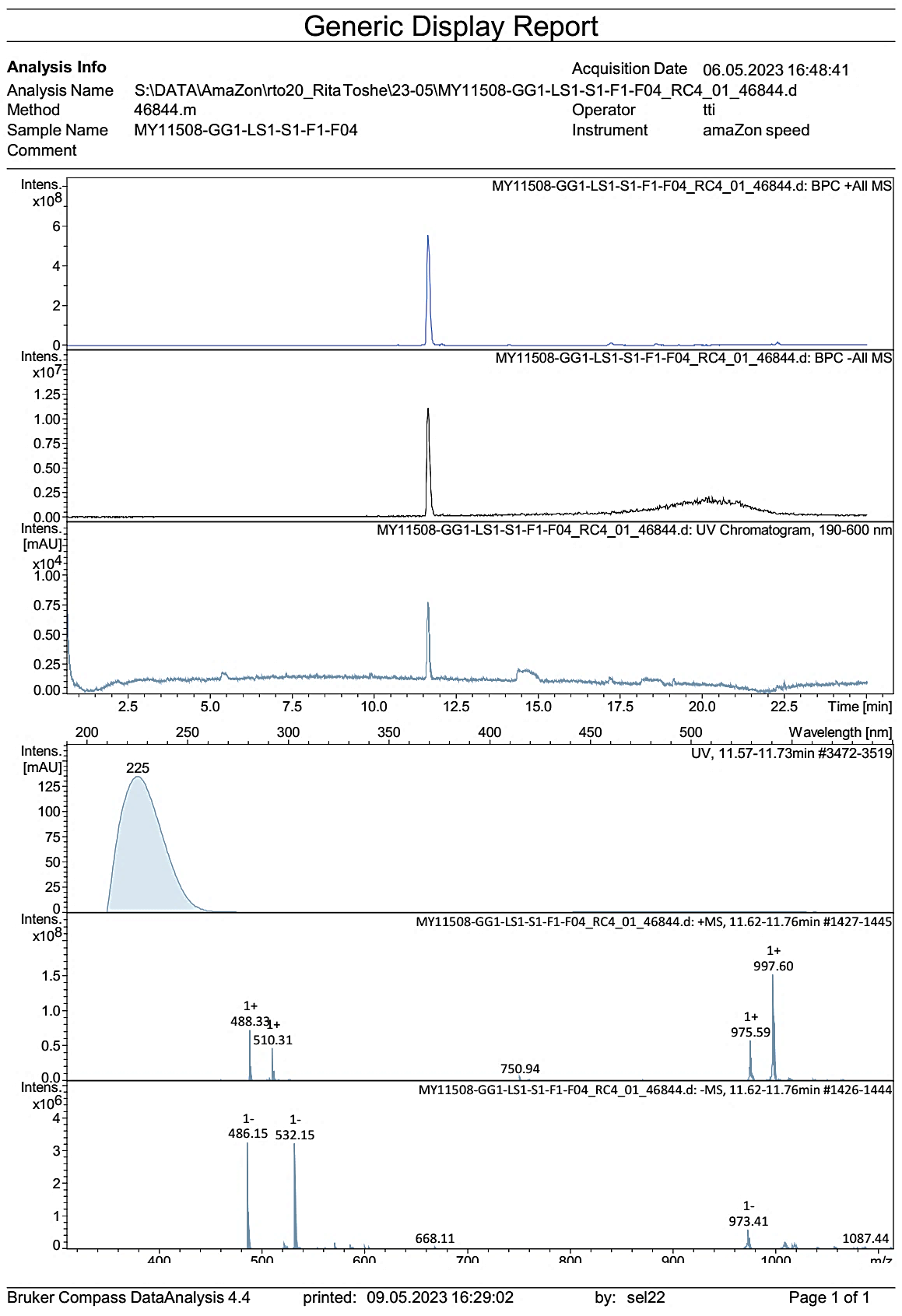


**11.75**

[2M+Na]^+^

[2M-H]^-^

[2M+H]^+^

[M-H]^-^

[M+H]^+^

[M+Na]^+^

Figure S16. LR-ESI-MS spectrum of **4**.


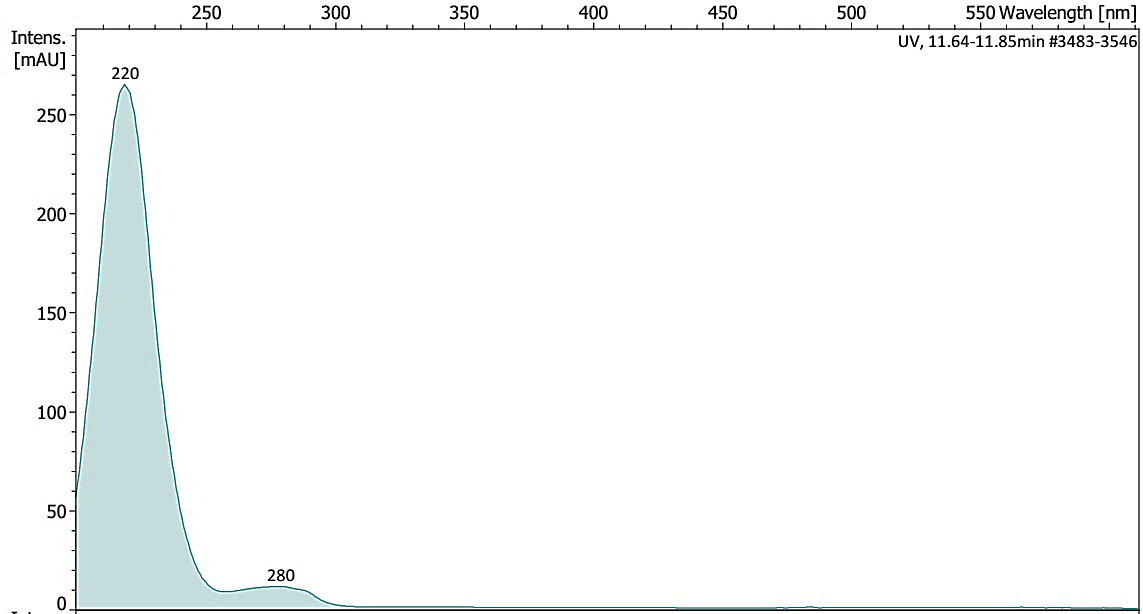

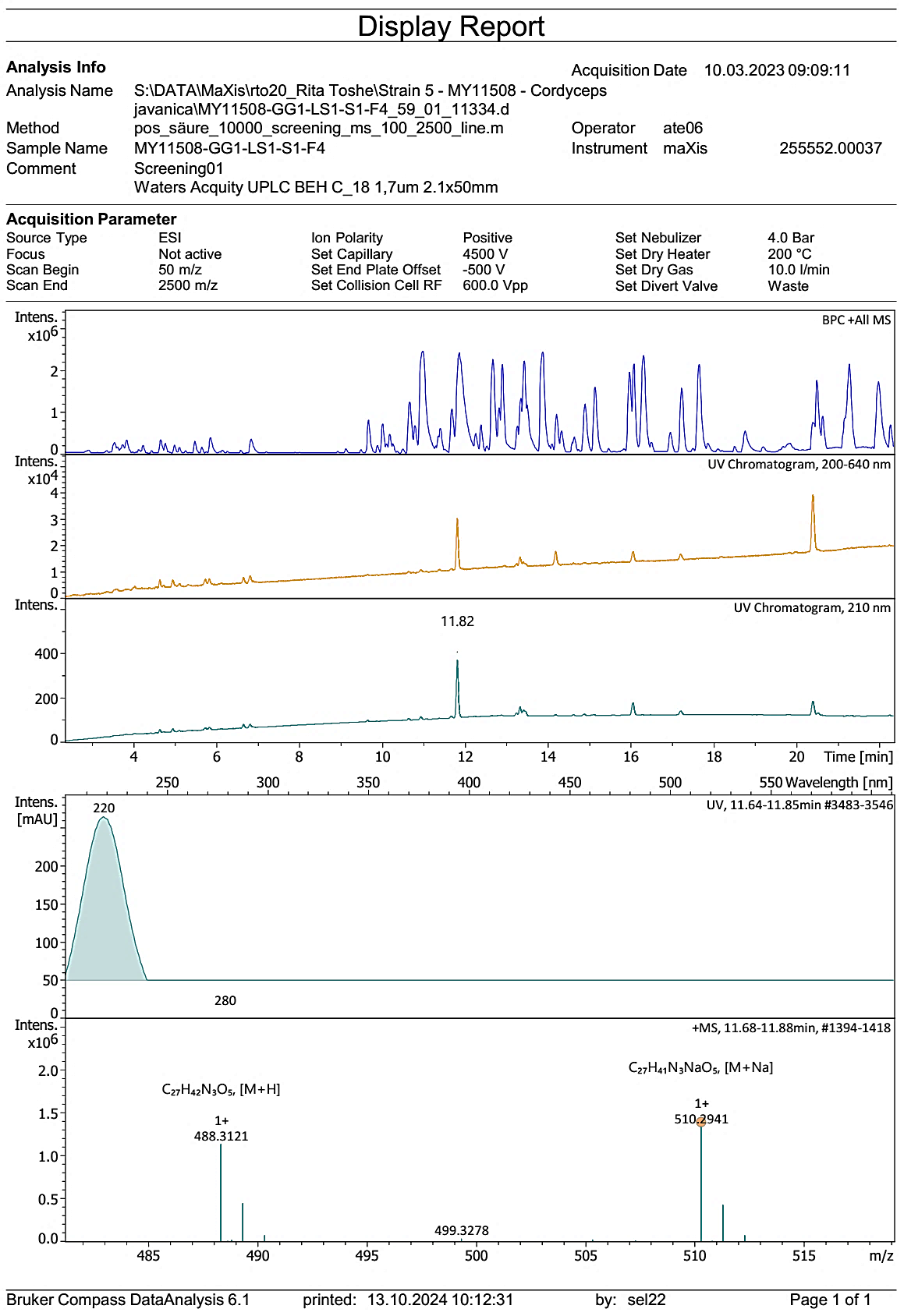


Figure S17. HR-ESI-MS spectrum of **4**.

Figure S18. ^1^H NMR spectrum of **4** in DMSO-*d*_6_ at 500 MHz.

Figure S19. ^1^H–^1^H COSY spectrum of **4** in DMSO-*d*_6_ at 500 MHz.


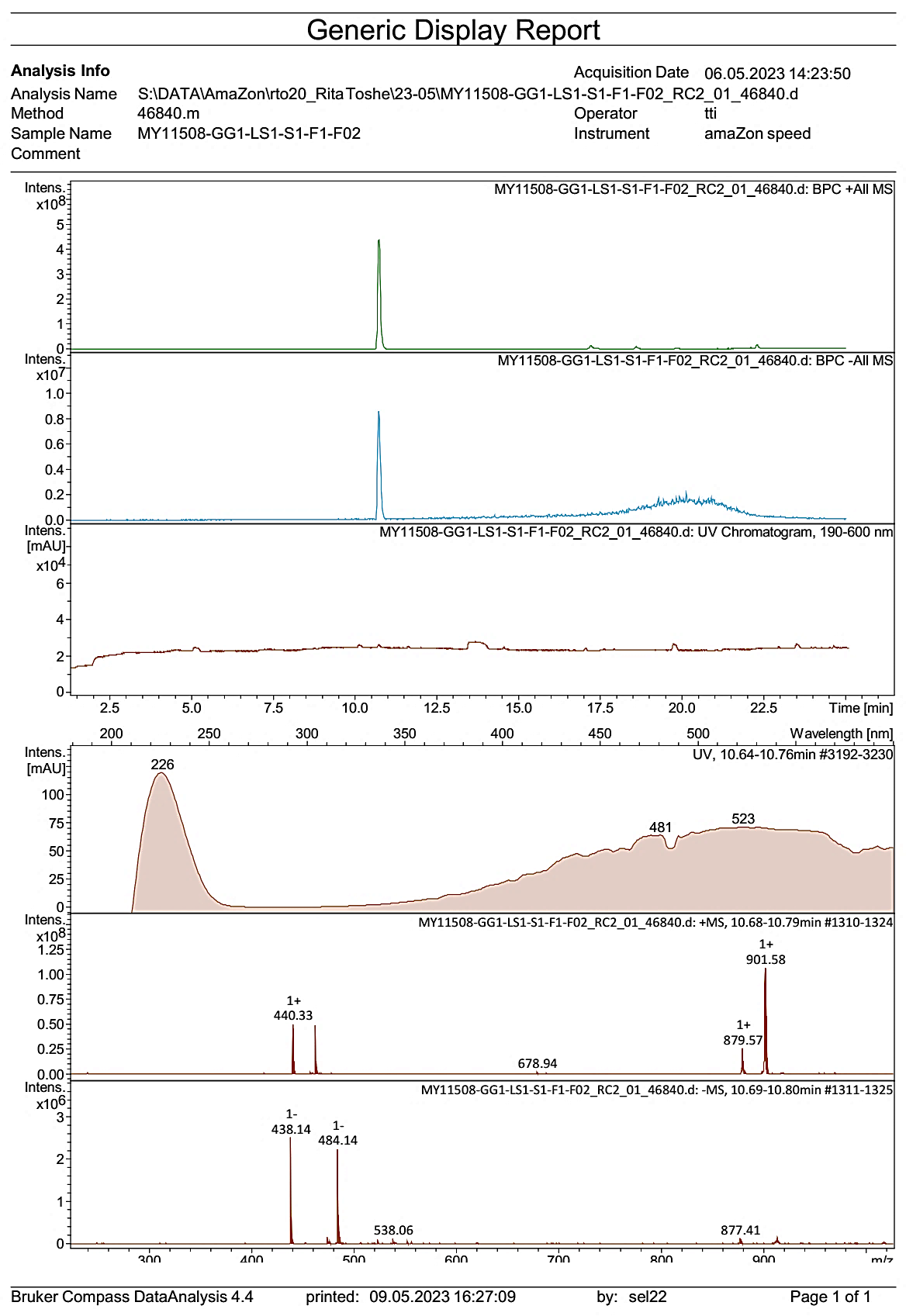


[2M-H]^-^

[2M+Na]^+^

[2M+H]^+^

[M+H]^+^

[M-H]^-^

[M+Na]^+^

**10.74**

**1+**

**462.33**

Figure S20. LR-ESI-MS spectrum of **5**.

Figure S21. ^1^H NMR spectrum of **5** in DMSO-*d*_6_ at 500 MHz.

Figure S22. ^1^H–^1^H COSY spectrum of **5** in DMSO-*d*_6_ at 500 MHz.


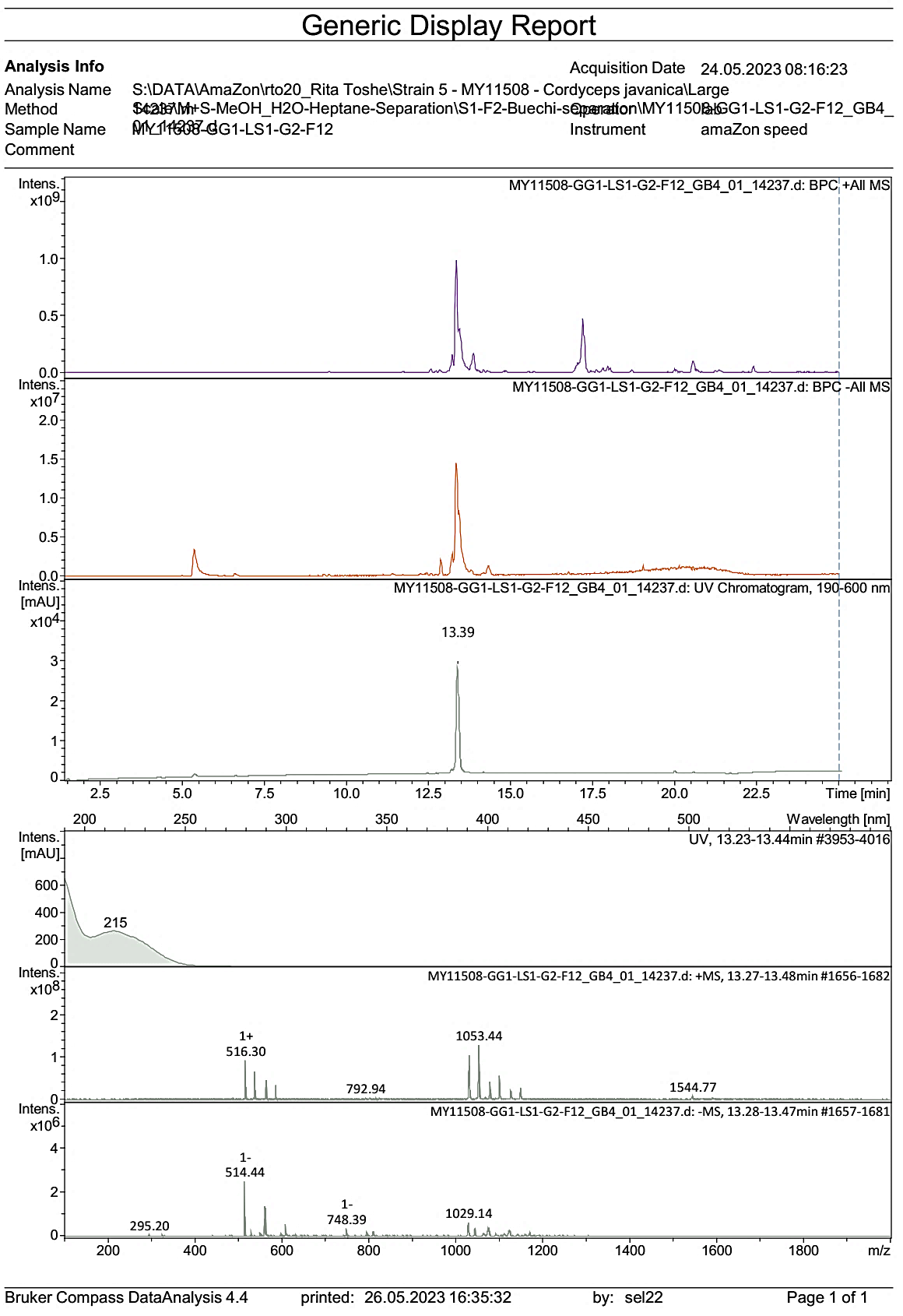


[2M-H]^-^

[2M+Na]^+^

[M+H]^+^

[M-H]^-^

Figure S23. LR-ESI-MS spectrum of **6**.


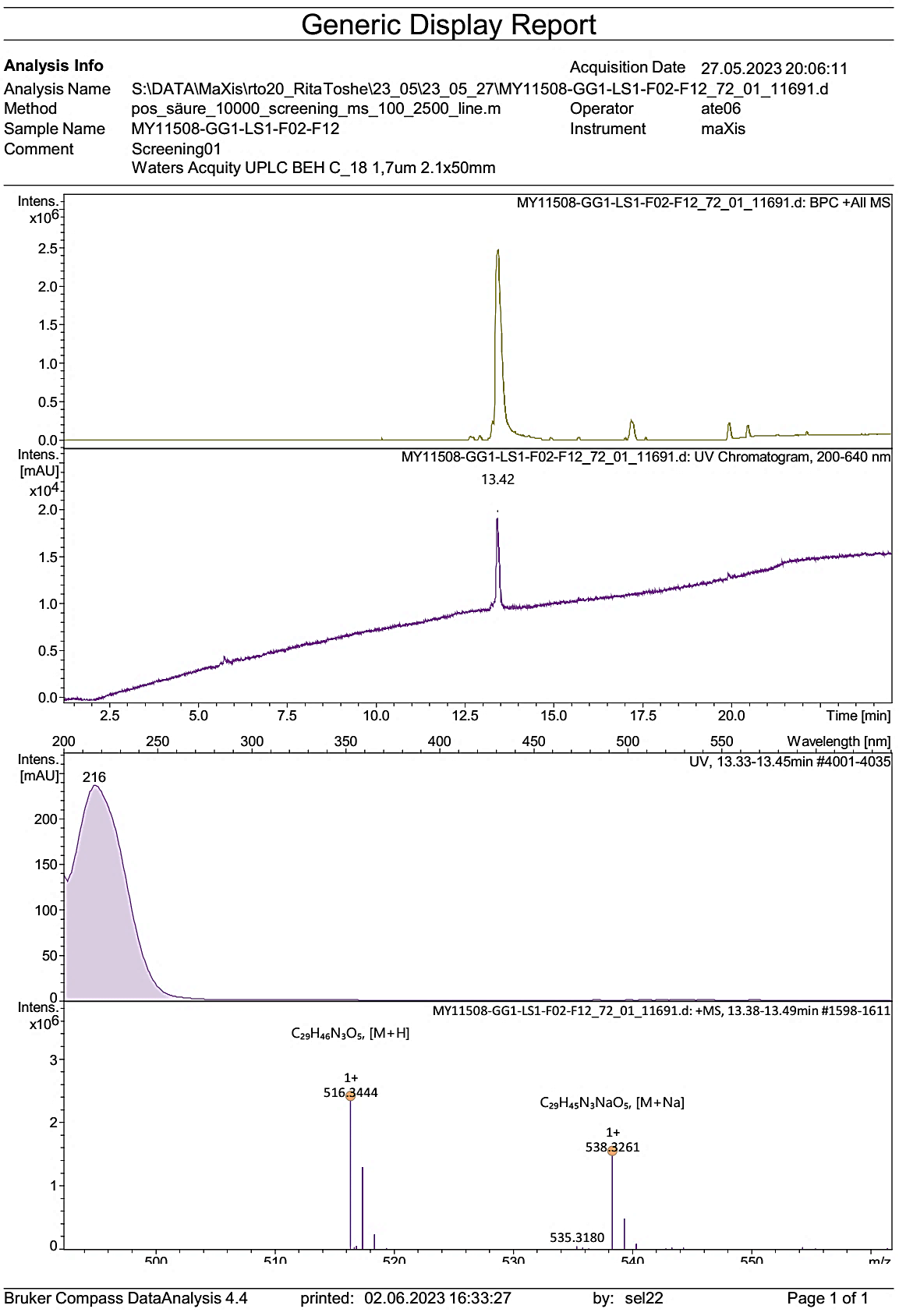


Figure S24. HR-ESI-MS spectrum of **6**.

Figure S25. ^1^H NMR spectrum of **6** in DMSO-*d*_6_ at 500 MHz.


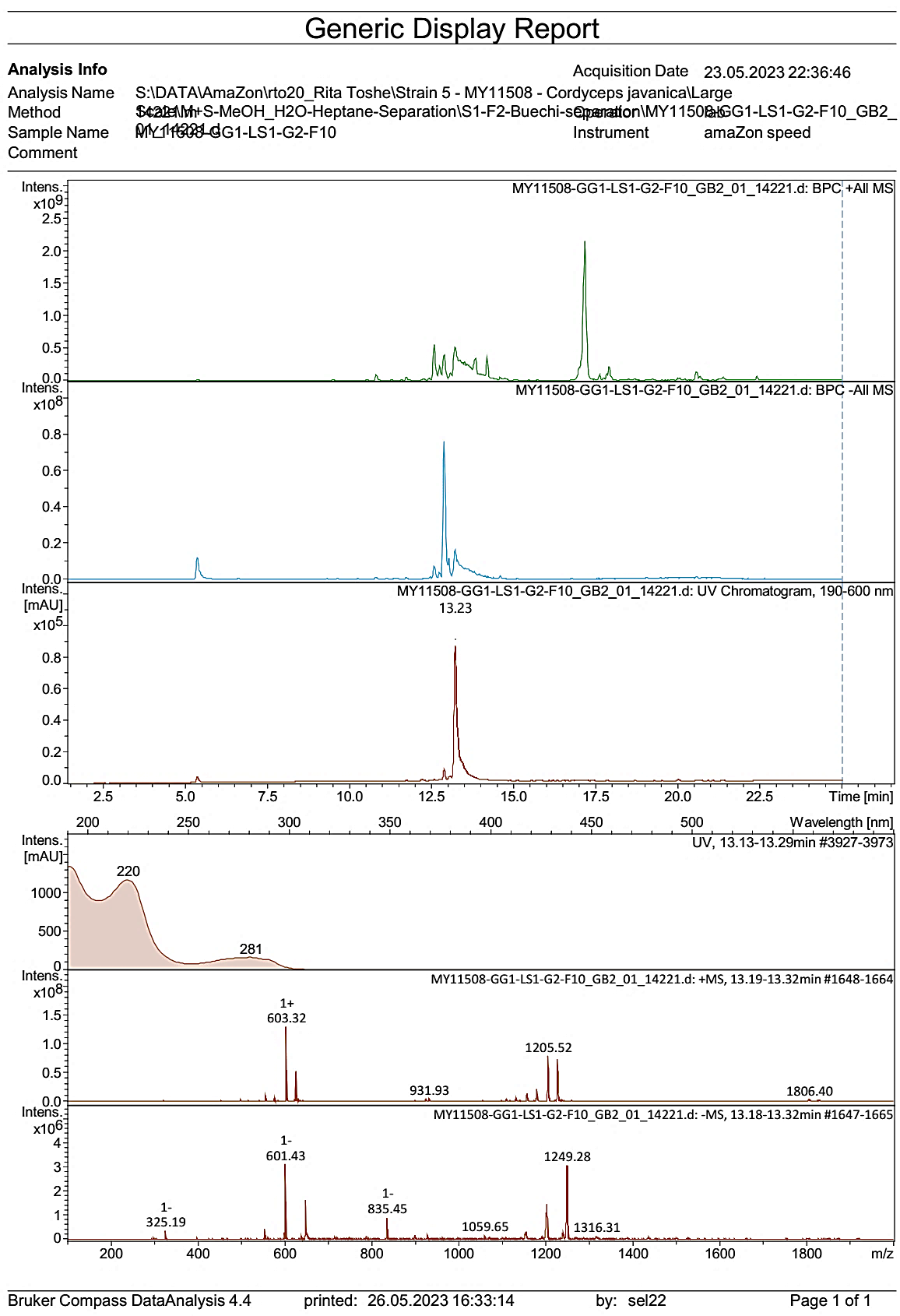


[M-H]^-^

[2M+H]^+^

[M+H]^+^

Figure S26. LR-ESI-MS spectrum of **7**.


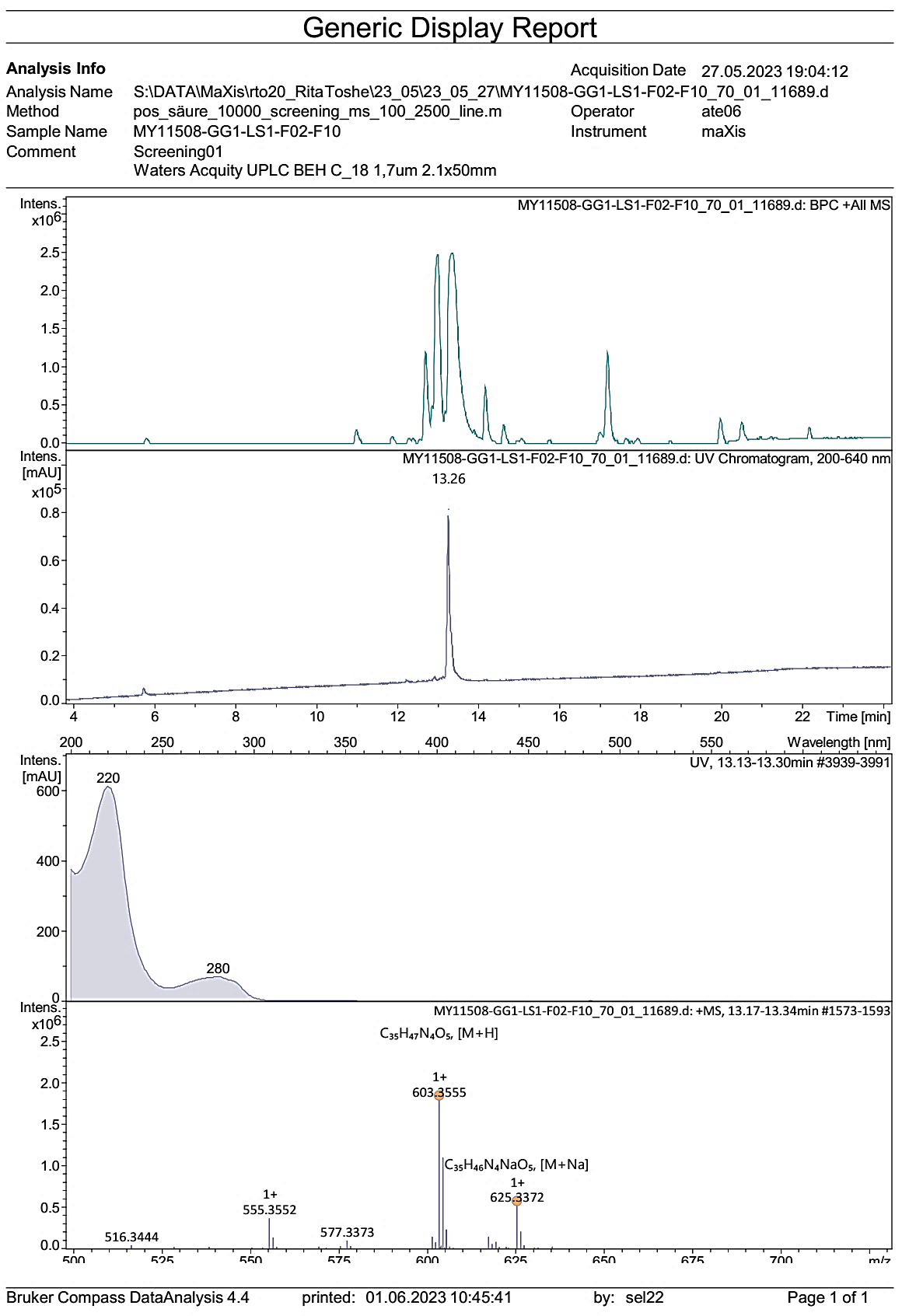


Figure S27. HR-ESI-MS spectrum of **7**.

Figure S28. ^1^H NMR spectrum of **7** in DMSO-*d*_6_ at 500 MHz.

Figure S29. ^1^H–^1^H COSY spectrum of **7** in DMSO-*d*_6_ at 500 MHz.

Figure S30. HMBC spectrum of **7** in DMSO-*d*_6_ at 500 MHz.

Figure S31. HSQC spectrum of **7** in DMSO-*d*_6_ at 500 MHz.


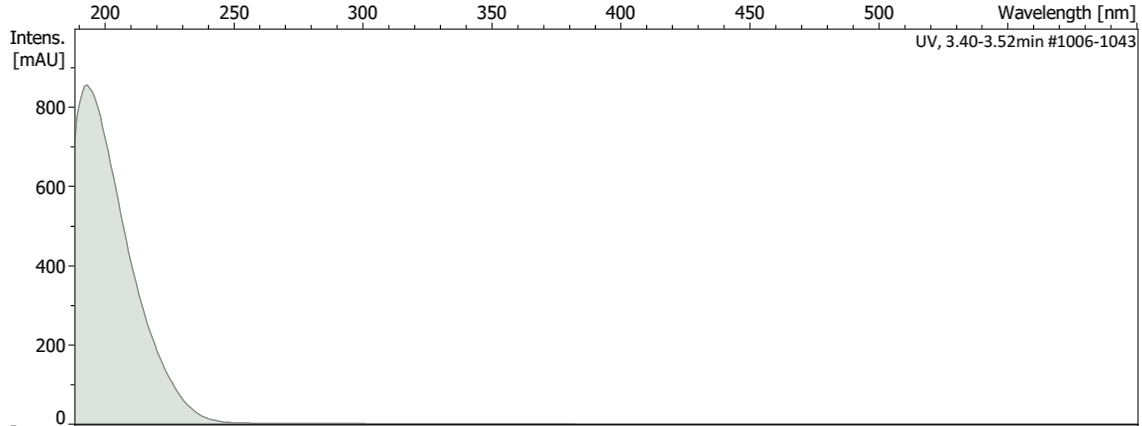

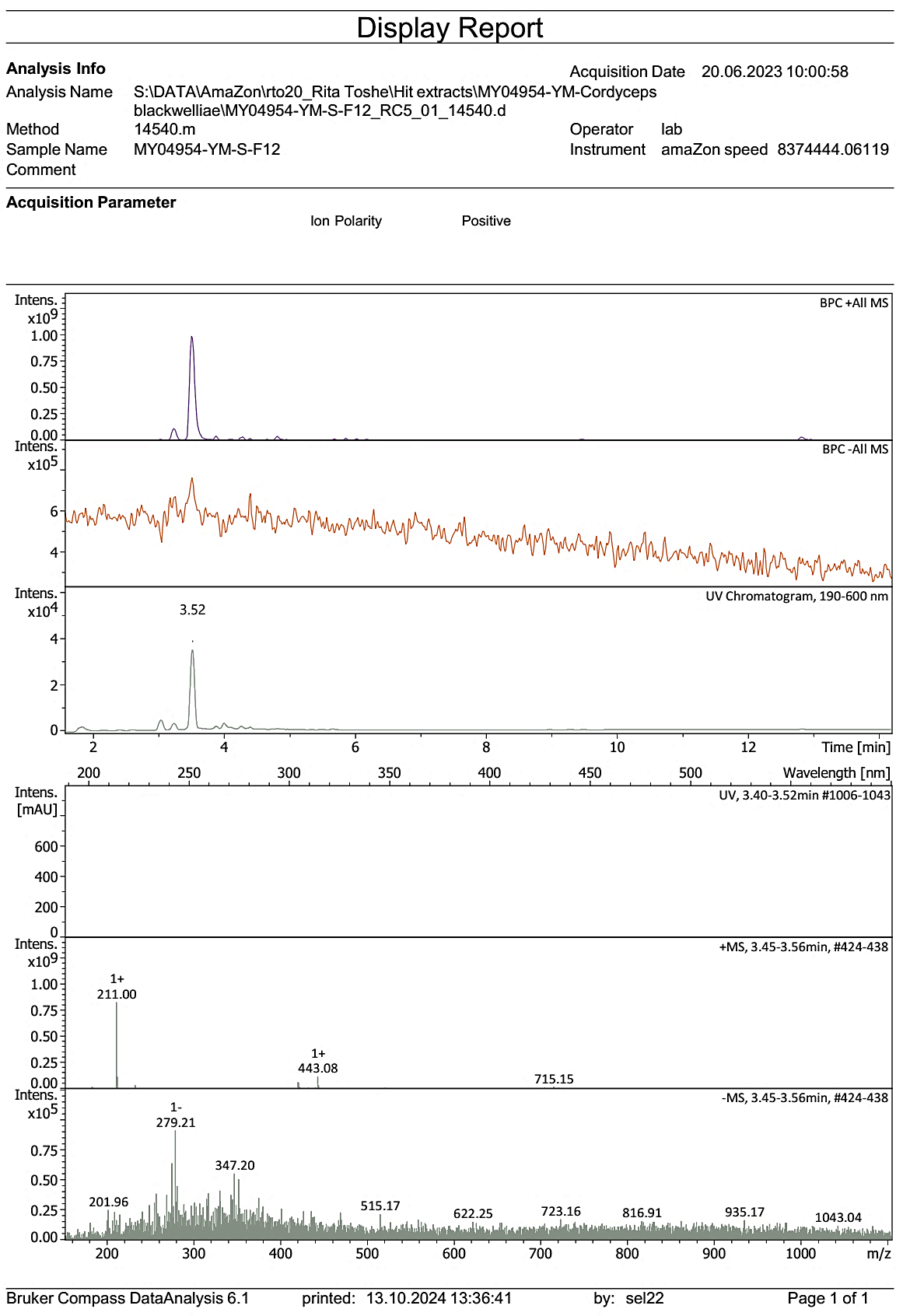


[2M+Na]^+^

[M+H]^+^

Figure S32. LR-ESI-MS spectrum of **8**.


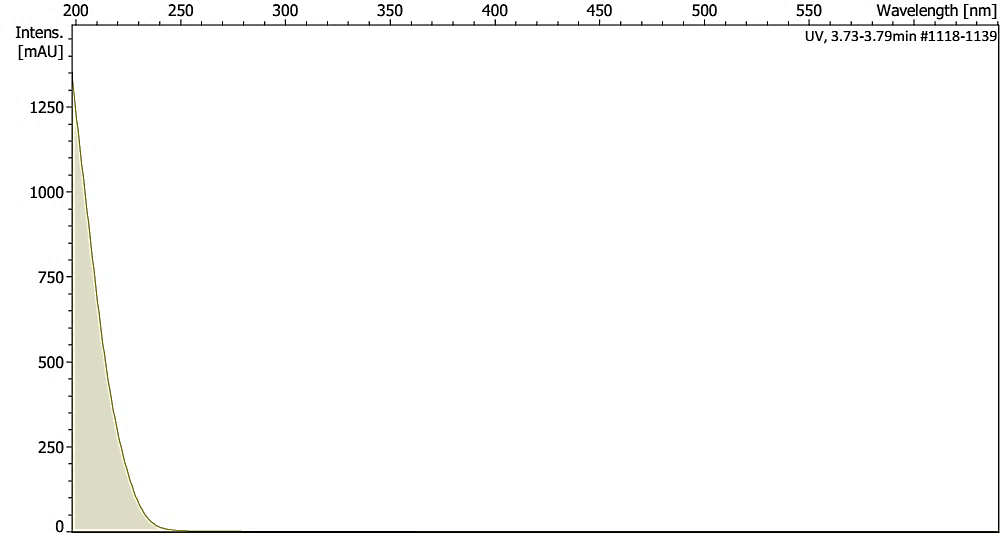

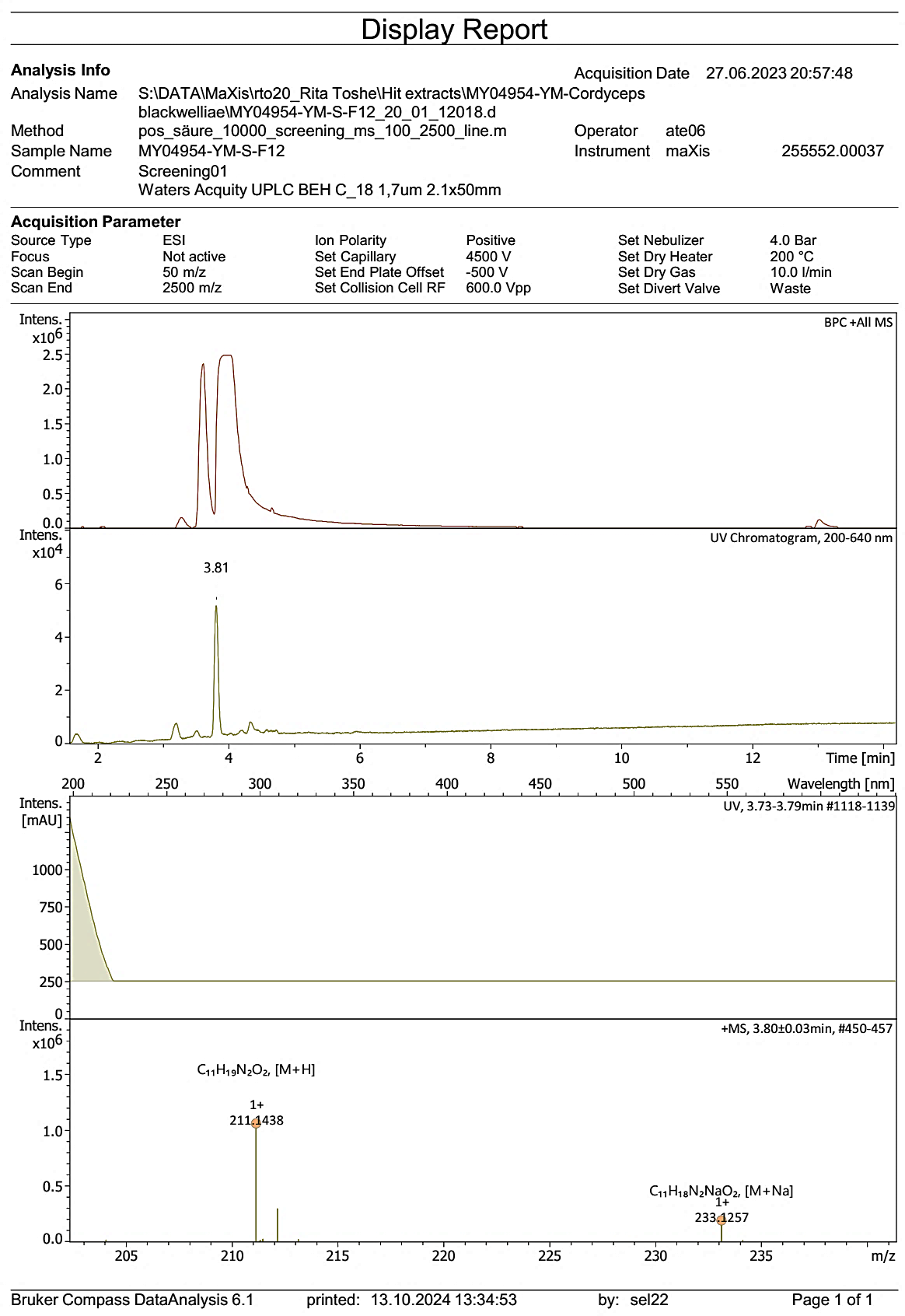


Figure S33. HR-ESI-MS spectrum of **8**.

Figure S34. ^1^H NMR spectrum of **8** in DMSO-*d*_6_ at 500 MHz.


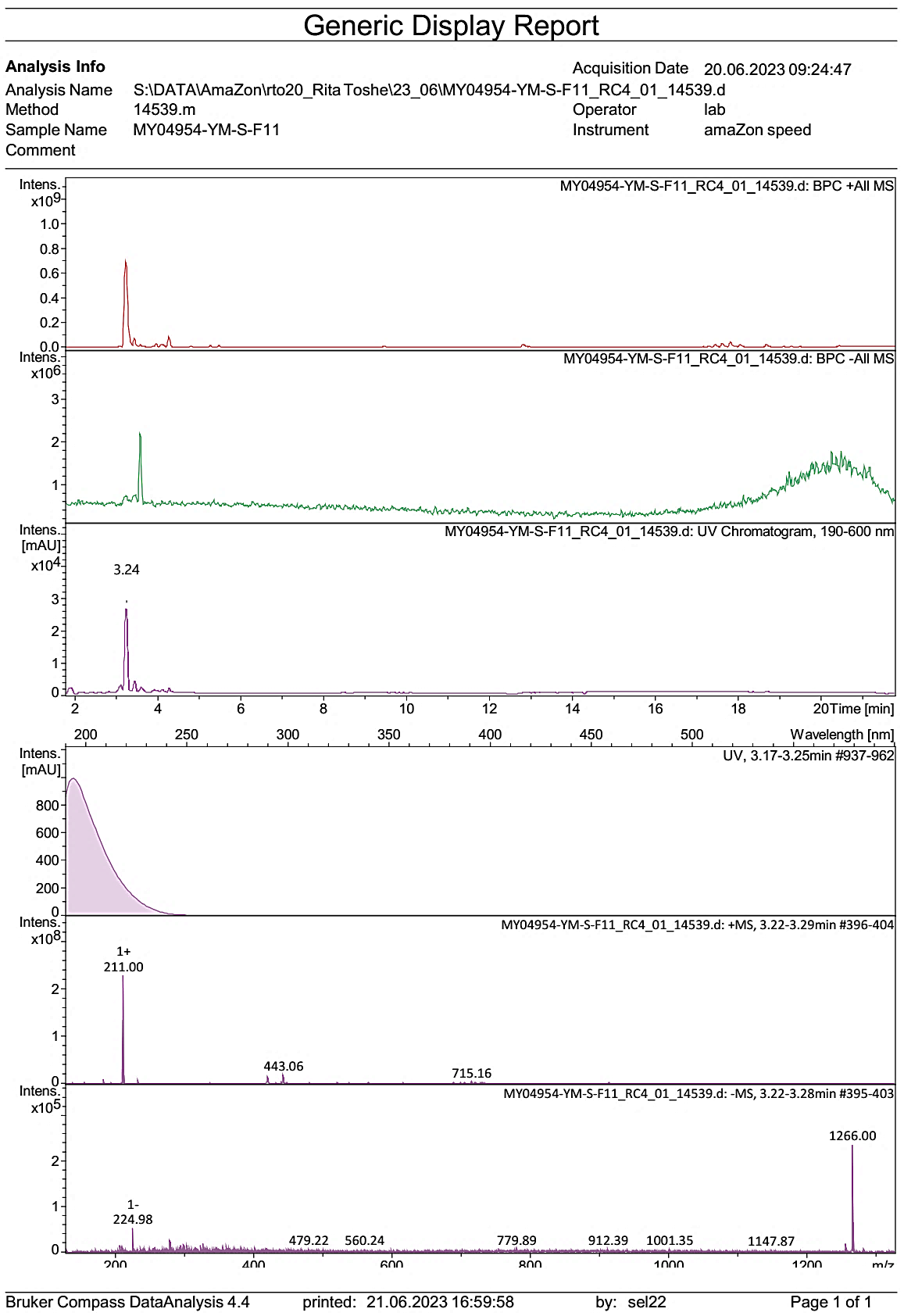


[M+H]^+^

[2M+Na]^+^

Figure S35. LR-ESI-MS spectrum of **9**.


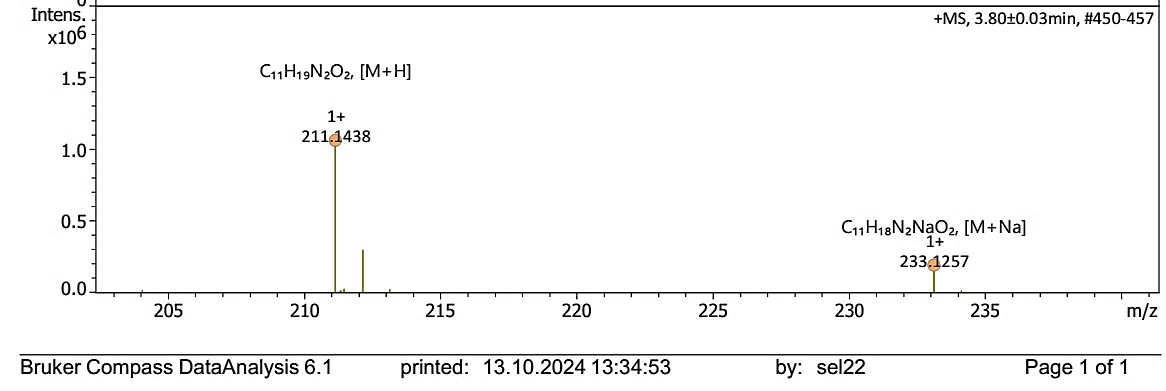

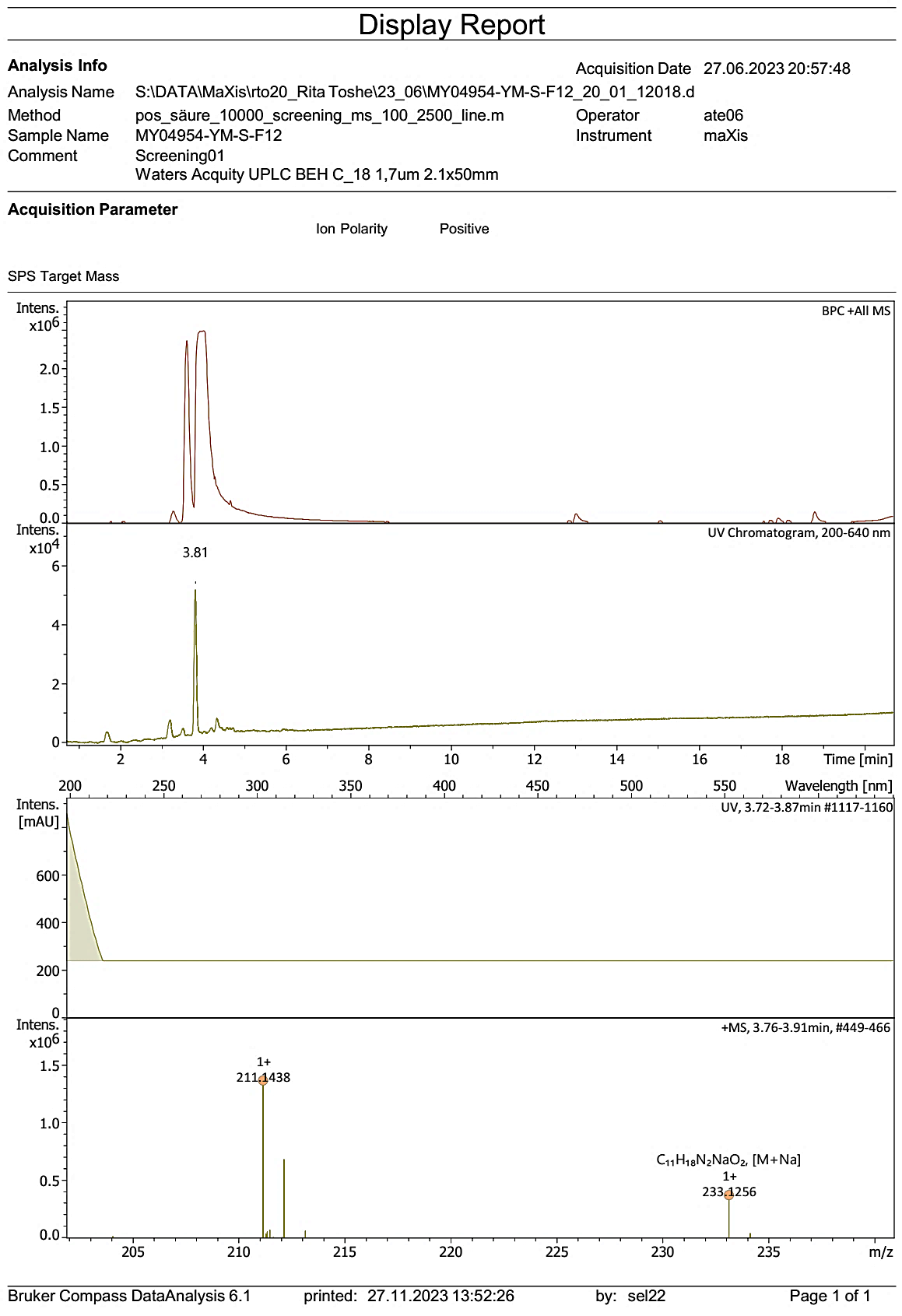


Figure S36. HR-ESI-MS spectrum of **9**.

Figure S37. ^1^H NMR spectrum of **9** in DMSO-*d*_6_ at 500 MHz.

Figure S38. ^13^C NMR spectrum of **9** in DMSO-*d*_6_ at 125 MHz.

Figure S39. ^1^H–^1^H COSY spectrum of **9** in DMSO-*d*_6_ at 500 MHz.

Figure S40. HMBC spectrum of **9** in DMSO-*d*_6_ at 500 MHz.

Figure S41. HSQC spectrum of **9** in DMSO-*d*_6_ at 500 MHz.

Figure S42. ROESY spectrum of **9** in DMSO-*d*_6_ at 500 MHz.


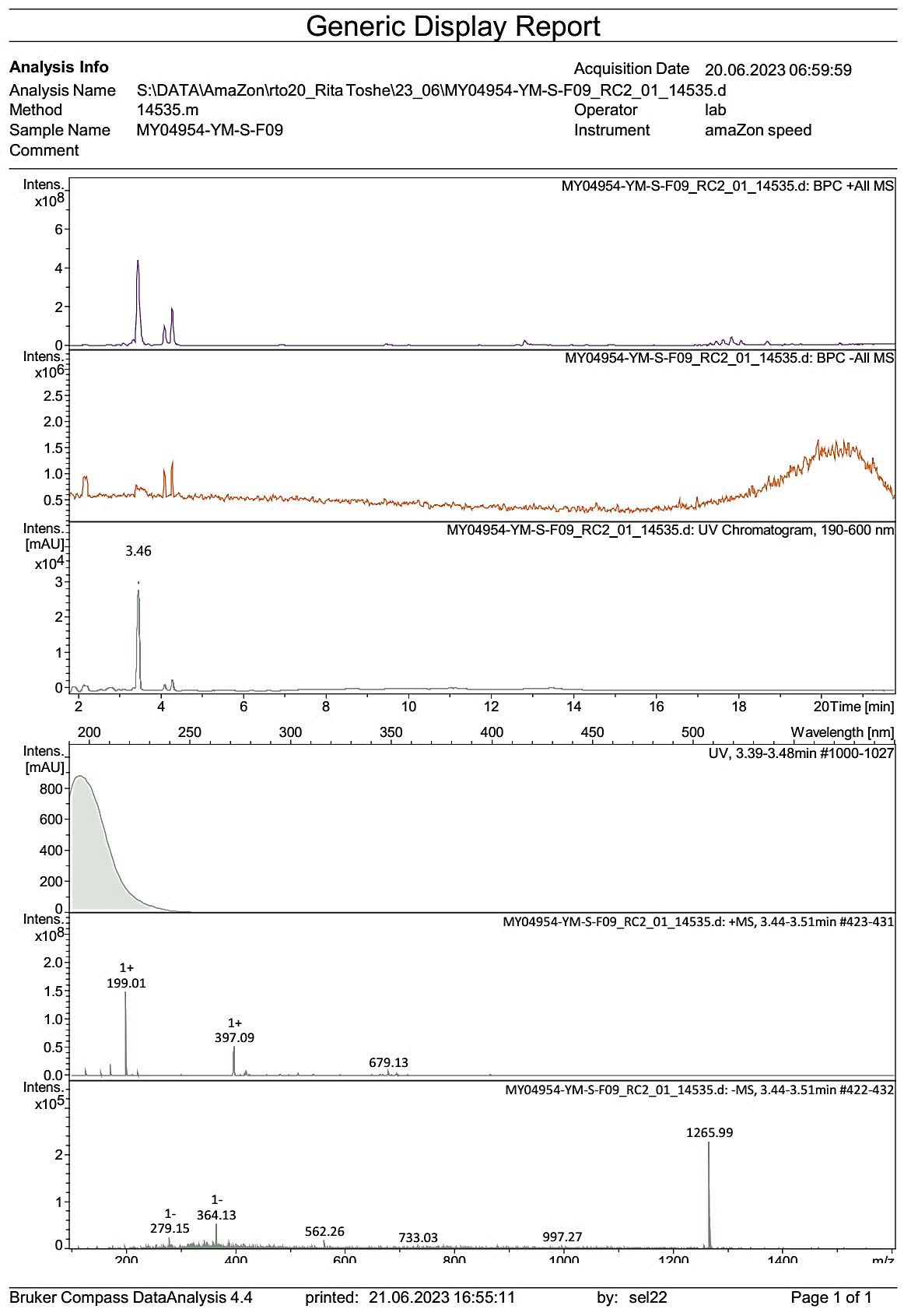


[M+H]^+^

[2M+Na]^+^

Figure S43. LR-ESI-MS spectrum of **10**.


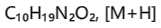

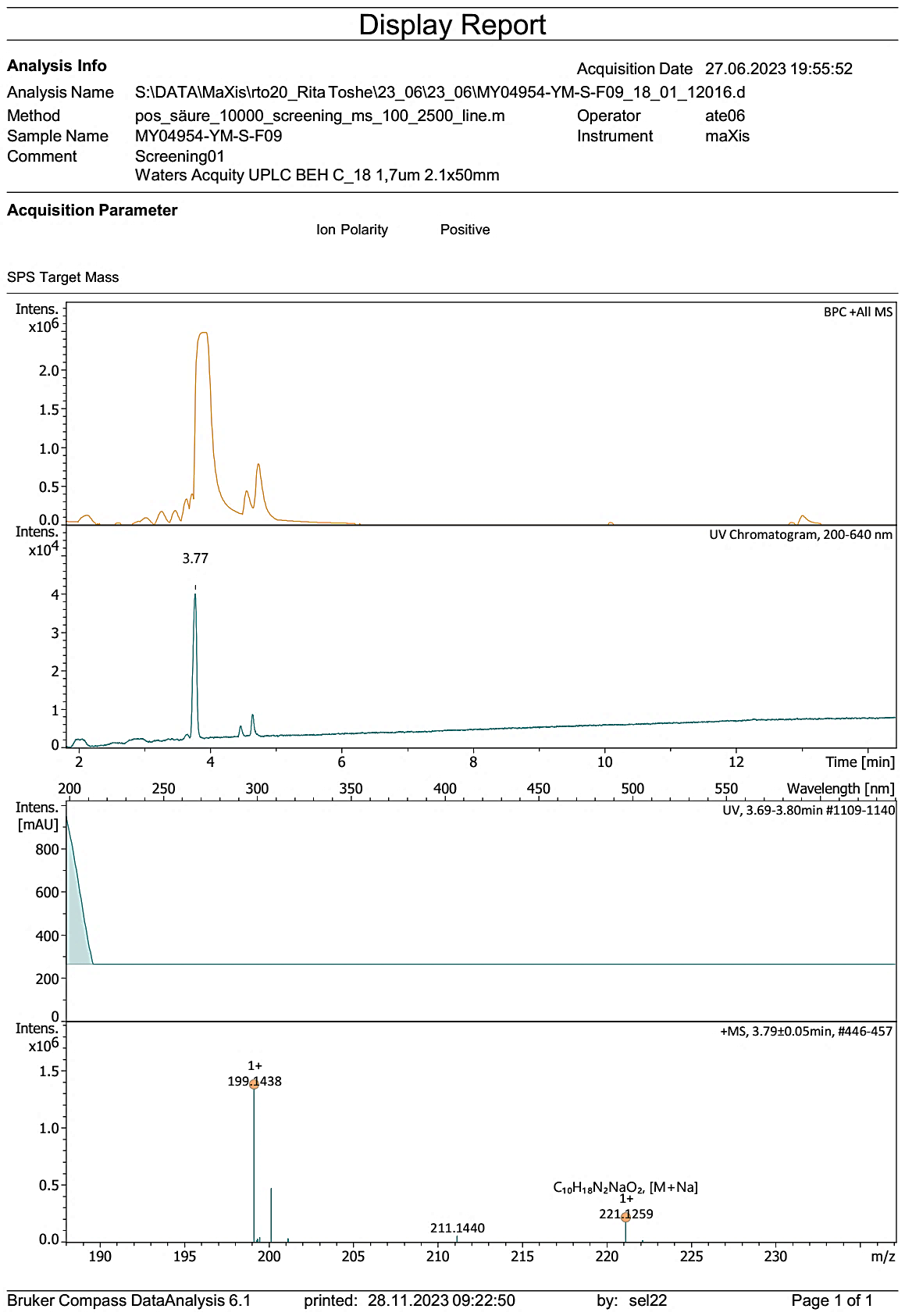


Figure S44. HR-ESI-MS spectrum of **10**.

Figure S45. ^1^H NMR spectrum of **10** in DMSO-*d*_6_ at 500 MHz.

Figure S46. ^1^H–^1^H COSY spectrum of **10** in DMSO-*d*_6_ at 500 MHz.

Figure S47. HMBC spectrum of **10** in DMSO-*d*_6_ at 500 MHz.

Figure S48. ROESY spectrum of **10** in DMSO-*d*_6_ at 500 MHz.


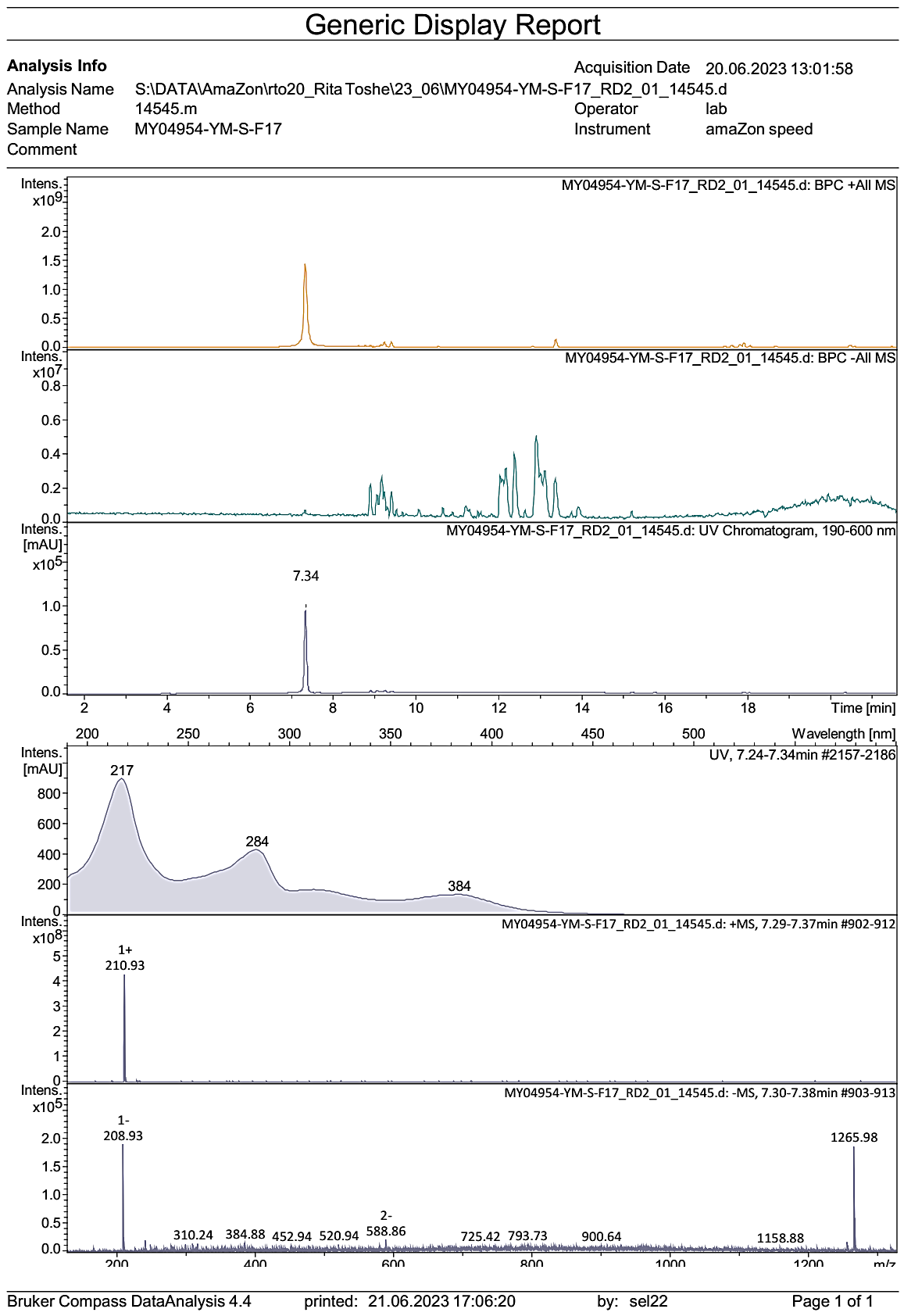


[M-H]^-^

[M+H]^+^

Figure S49. LR-ESI-MS spectrum of **11**.

Figure S50. ^1^H NMR spectrum of **11** in DMSO-*d*_6_ at 500 MHz.

Figure S51. DEPTQ spectrum of **11** in DMSO-*d*_6_ at 125 MHz.

Figure S52. ^1^H–^1^H COSY spectrum of **11** in DMSO-*d*_6_ at 500 MHz.

Figure S53. HMBC spectrum of **11** in DMSO-*d*_6_ at 500 MHz.

Figure S54. HSQC spectrum of **11** in DMSO-*d*_6_ at 500 MHz.

Figure S55. ROESY spectrum of **11** in DMSO-*d*_6_ at 500 MHz.


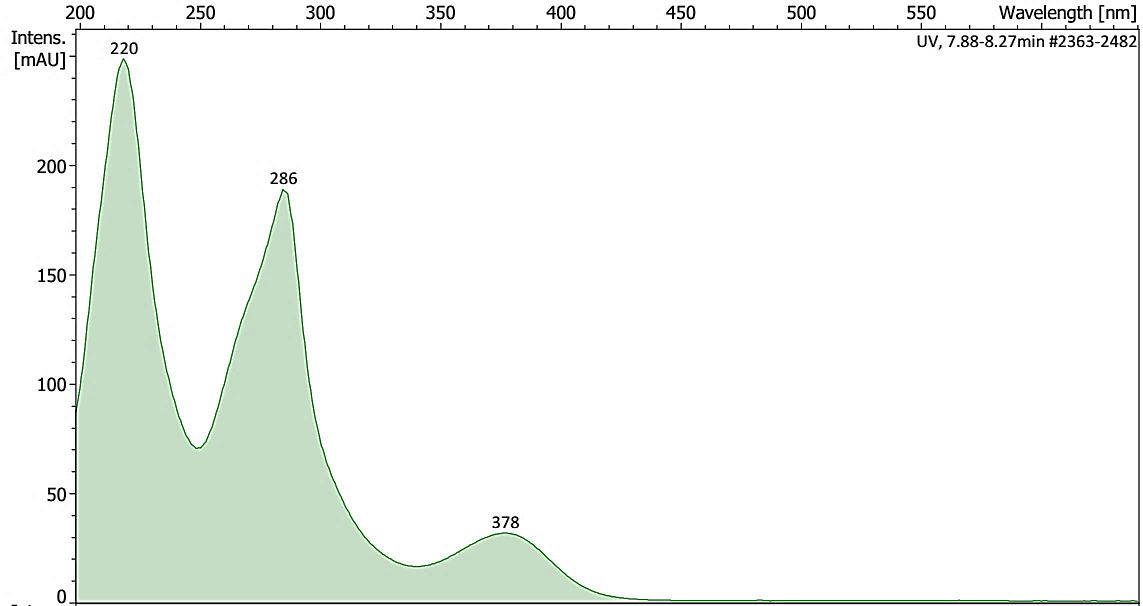

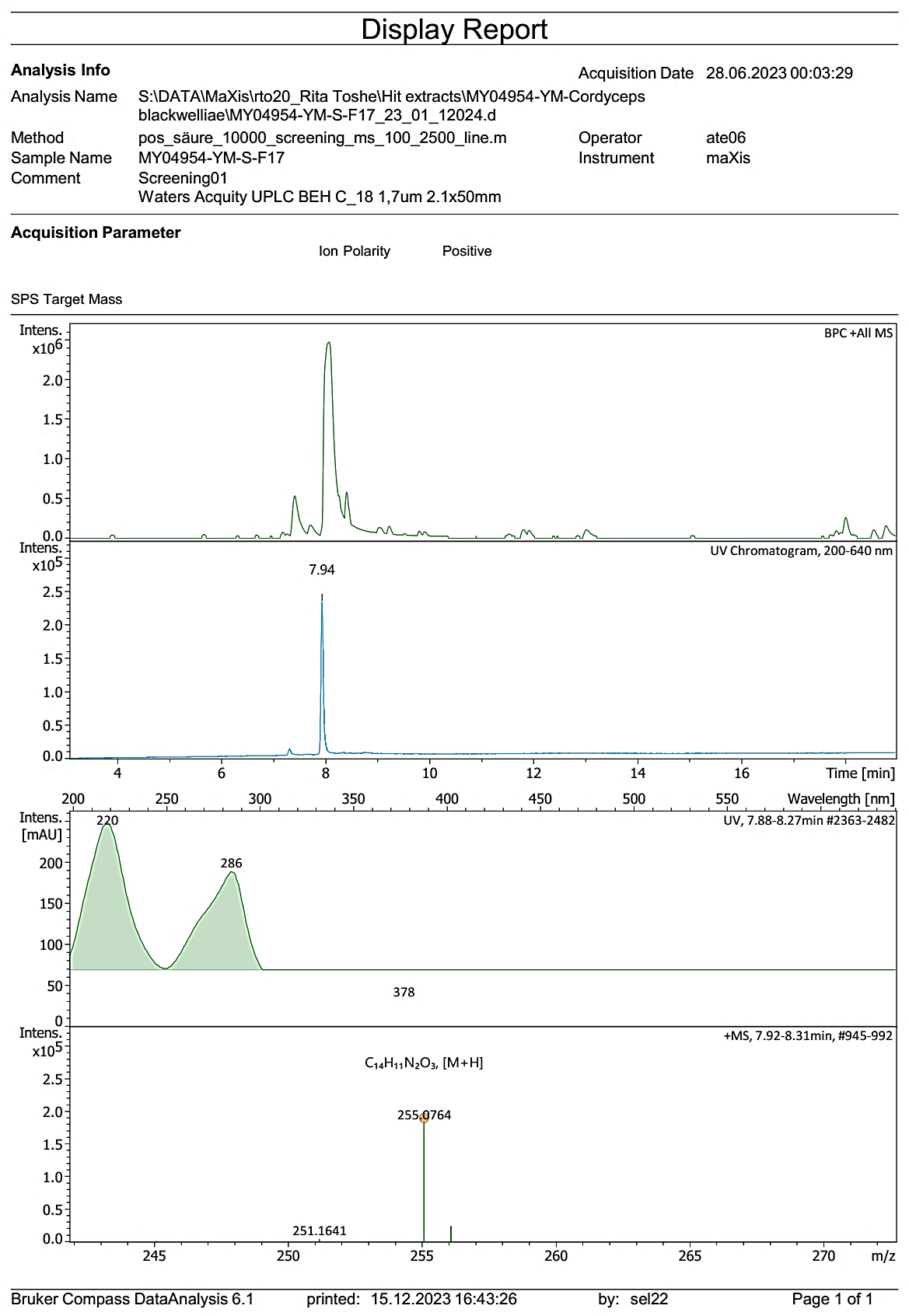


Figure S56. HR-ESI-MS spectrum of **12**.

Figure S57. ^1^H NMR spectrum of **12** in DMSO-*d*_6_ at 500 MHz.

Figure S58. ^1^H–^1^H COSY spectrum of **12** in DMSO-*d*_6_ at 500 MHz.

Figure S59. HMBC spectrum of **12** in DMSO-*d*_6_ at 500 MHz.

Figure S60. HSQC spectrum of **12** in DMSO-*d*_6_ at 500 MHz.

Table S3. Antimicrobial properties of the isolated metabolites.

| **Test Microorganism** | **MIC (µg/mL)** | | | | | | | | | | | | **Positive Control (µg/mL)** |
| --- | --- | --- | --- | --- | --- | --- | --- | --- | --- | --- | --- | --- | --- |
|  | **1** | **2** | **3** | **4** | **5** | **6** | **7** | **8** | **9** | **10** | **11** | **12** |  |
| *Staphylococcus aureus* (DSM 346) | n.i. | n.i. | 66.6 | 66.6 | n.i. | n.i. | *n.i.* | n.i. | n.i. | n.i. | n.i. | n.i. | 0.21^G^ |
| *Escherichia coli* (DSM 1116) | n.i. | n.i. | n.i. | n.i. | n.i. | n.i. | n.i. | n.i. | n.i. | n.i. | n.i. | n.i. | 0.42^G^ |
| *Bacillus subtilis* (DSM 10) | n.i. | n.i. | n.i. | 66.6 | n.i. | n.i. | n.i. | n.i. | n.i. | n.i. | n.i. | n.i. | 16.6^O^ |
| *Pseudomonas aeruginosa* (PA14) | n.i. | n.i. | n.i. | n.i. | n.i. | n.i. | n.i. | n.i. | n.i. | n.i. | n.i. | n.i. | 0.21^G^ |
| *Wickerhamomyces anomalus* (DSM 6766) | n.i. | n.i. | n.d. | n.d. | n.d. | n.d. | n.i. | n.i. | n.i. | n.d. | n.d. | n.i. | 16.6^N^ |
| *Candida albicans* (DSM 1665) | n.i. | n.i. | n.i. | n.i. | n.i. | n.i. | n.i. | n.i. | n.i. | n.i. | n.i. | n.i. | 8.3^N^ |
| *Acinetobacter baumannii* (DSM 30008) | n.i. | n.i. | n.d. | n.d. | n.d. | n.d. | n.i. | n.i. | n.i. | n.d. | n.d. | n.i. | 0.52^C^ |
| *Chromobacterium violaceum* (DSM 30191) | n.i. | n.i. | n.d. | n.d. | n.d. | n.d. | n.i. | n.i. | n.i. | n.d. | n.d. | n.i. | 1.70^G^ |
| *Schizosaccharomyces pombe* (DSM 70572) | n.i. | n.i. | n.d. | n.d. | n.d. | n.d. | n.i. | n.i. | n.i. | n.d. | n.d. | n.i. | 8.30^N^ |
| *Mucor hiemalis* (DSM 2656) | n.i. | n.i. | n.i. | n.i. | 66.6 | n.i. | n.i. | n.i. | n.i. | n.i. | n.i. | n.i. | 8.30^N^ |
| *Rhodotorula glutinis* (DSM 10134) | n.i. | n.i. | n.d. | n.d. | n.d. | n.d. | n.i. | n.i. | n.i. | n.d. | n.d. | n.i. | 4.20^N^ |
| *Mycobacterium smegmatis* (ATCC 700084) | n.i. | n.i. | n.i. | n.i. | n.i. | n.i. | n.i. | n.i. | n.i. | n.i. | n.i. | n.i. | 1.70^K^ |

n.i.: No inhibition up to 67 µg/mL. n.d.: Not determined. G: gentamicin; O: oxytetracycline; N: nystatin; C: ciprofloxacin; K: kanamycin.

Table S4. Cytotoxic properties of the isolated metabolites.

| **Test Cell Line** | **IC_50_ (µM)** | | | | | | | | | | | | **Positive Control** |
| --- | --- | --- | --- | --- | --- | --- | --- | --- | --- | --- | --- | --- | --- |
|  | **1** | **2** | **3** | **4** | **5** | **6** | **7** | **8** | **9** | **10** | **11** | **12** | **Epothilone B (nM)** |
| Mouse fibroblast (L929) | n.a. | n.a. | n.a. | n.a. | n.a. | n.a. | n.a. | n.a. | n.a. | n.a. | n.a. | n.a. | 0.65 |
| Human endocervival adenocarcinoma (KB3.1) | n.a. | n.a. | n.a. | 13.3 | n.a. | n.a. | 36.5 | n.a. | n.a. | n.a. | n.a. | n.a. | 0.17 |
| Human prostate carcinoma (PC-3) | n.d. | n.d. | n.d. | 3.5 | n.d. | n.d. | n.d. | n.d. | n.d. | n.d. | n.d. | n.d. | 0.09 |
| Human breast adenocarcinoma (MCF-7) | n.d. | n.d. | n.d. | n.a. | n.d. | n.d. | n.d. | n.d. | n.d. | n.d. | n.d. | n.d. | 0.07 |
| Human ovarian cancer (SKOV-3) | n.d. | n.d. | n.d. | n.a. | n.d. | n.d. | n.d. | n.d. | n.d. | n.d. | n.d. | n.d. | 0.09 |
| Human epidermoid carcinoma (A431) | n.d. | n.d. | n.d. | 1.2 | n.d. | n.d. | n.d. | n.d. | n.d. | n.d. | n.d. | n.d. | 0.06 |
| Human lung carcinoma (A549) | n.d. | n.d. | n.d. | 0.9 | n.d. | n.d. | n.d. | n.d. | n.d. | n.d. | n.d. | n.d. | 0.05 |

n.a.: Not active. n.d.: Not determined.
